# Progress in Additive Manufacturing of High-Entropy Alloys

**DOI:** 10.3390/ma17235917

**Published:** 2024-12-03

**Authors:** Bin Chen

**Affiliations:** School of Materials Science and Engineering, Shanghai Jiao Tong University, Shanghai 200240, China; steelboy@sjtu.edu.cn

**Keywords:** additive manufacturing, high-entropy alloys, microstructure, mechanical properties, corrosion performances

## Abstract

High-entropy alloys (HEAs) have drawn substantial attention on account of their outstanding properties. Additive manufacturing (AM), which has emerged as a successful approach for fabricating metallic materials, allows for the production of complex components based on three-dimensional (3D) computer-aided design (CAD) models. This paper reviews the advancements in the AM of HEAs, encompassing a variety of AM techniques, including selective laser melting (SLM), selective laser sintering (SLS), selective electron beam melting (SEBM), directed energy deposition (DED), binder jetting (BJT), direct ink writing (DIW), and additive friction stir deposition (AFSD). Additionally, the study discusses the powders and wires utilized in AM, the post-processing of AM-processed HEAs, as well as the mechanical and corrosion properties of these alloys. The unique ultra-fine and non-equilibrium microstructures achieved through AM result in superior mechanical properties of HEAs, like improved strength and ductility. However, research regarding certain aspects of HEA AM, such as fatigue properties and creep deformation behavior, is still relatively scarce. Future research should focus on overcoming the existing limitations and exploring the potential of HEAs in various applications.

## 1. Introduction

### 1.1. Introduction of High-Entropy Alloys

In recent years, high-entropy alloys (HEAs) have received great attention due to their features. Such features involve the combination of high strength and ductility; microstructural stability; retained mechanical strength at elevated temperatures; as well as resistance to wear, fatigue, corrosion, and oxidation.

The precursor to research on HEAs can be traced back to the German scientist Franz Karl Archard [1]. In the 18th century, Archard conducted investigations on equi-mass multicomponent alloys containing between five to seven elements, including Fe, Sn, Pb, Zn, Bi, Ag, Co, Sb, As, and Cu. His investigations encompassed the assessment of mechanical properties including ductility, hardness, impact resistance, wear resistance, and density. He published his research results in a French publication titled “Recherches sur les propriétés des alliages métalliques” [2]. Building on Archard’s pioneering work, Brian Cantor initiated research at the University of Sussex in England with his student Alain Vincent in 1981 [3], exploring a range of equimolar combinations consisting of 20 different elements (Mn, Cr, Fe, Co, Ni, Cu, Ag, W, Mo, Nb, Al, Cd, Sn, Pb, Bi, Zn, Ge, Si, Sb, Mg), and found that the CoCrFeMnNi alloy presented a single BCC structure. In 1996, J. W. Yeh developed various HEAs based on the concept that increasing entropy in an alloy system reduces the formation of multiple phases in the final product [4]. In 2003, S. Ranganathan authored a comprehensive review paper on the concept of HEAs, discussing the feasibility of fabricating these alloys [5]. The formal introduction of the concept of HEAs came in 2004 through two independent studies by Yeh et al. [6] and Cantor et al. [7]. Initially, HEAs were defined as alloys comprising five or more multi-principal elements, with approximately equi- or near equi-atomic ratios. Subsequently, researchers expanded the HEAs concept to include alloys containing three or four primary elements as well [8]. The term “High-Entropy Alloy” was initially defined by Yeh et al. [6] as alloys composed of five or more principal elements, each constituting between 5% to 35% of the alloy composition [9]. Yeh et al. [10] also proposed a second definition of HEA, categorizing HEAs into three types based on configurational entropy: low-entropy alloys (Δ*S_conf_* ≤ 0.69R), medium-entropy alloys (0.69R ≤ Δ*S_conf_* ≤ 1.61R), and HEAs (Δ*S_conf_* ≥ 1.61R) [11], where R is the universal gas constant. HEAs encompass compositions with five or more major elements, although equiatomic composition is not mandatory under this definition. HEAs once were typically limited to alloys with equal atomic compositions or single-phase microstructures. Due to the absence of a standardized definition, any metallic alloy containing multiple primary elements and exhibiting high configurational entropy is generally classified as an HEA.

The essential principle of HEAs centers on utilizing compositions featuring nearly equal proportions of five or more principal elements. As per the Gibbs phase rule, an augmentation in the number of constituent elements leads to a rise in the count of distinct phases. These phases predominantly consist of intermetallic compounds, given their significantly negative enthalpies. The fundamental principles within physical metallurgy and the empirical data derived from binary and ternary phase diagrams imply that employing multiple principal elements ought to result in the formation of numerous intermetallic compounds [12]. However, contrary to this expectation, it has been observed that HEAs possess a reduced number of phases. This reduction in phase complexity is due to the enhanced configurational entropy of HEAs. It promotes the formation of solid solutions instead of intermetallic phases, leading to less complex microstructures. A crucial key hypothesis suggests that the formation of highly random atomic configurations reduces the Gibbs free energy of the system, thus consequently thermodynamically suppressing the formation of intermetallic compounds [6].

Based on Boltzmann’s hypothesis related to the correlation of entropy and system complexity, during the formation of a solid solution from n elements with equimolar fractions, the change in configurational entropy per mole (∆*S_conf_*) can be calculated by using the following Equation (1) [9].

(1) $$\Delta S_{conf}=-k\ln w=-R\left(\frac{1}{n}\ln\frac{1}{n}+\frac{1}{n}\ln\frac{1}{n}+\cdots \frac{1}{n}\ln\frac{1}{n}\right)=-R\ln\frac{1}{n}=R\ln n$$

where *k* is Boltzmann’s constant, *w* is the number of ways of mixing, and *R* is the gas constant: 8.314 J/K mole.

Usually, HEAs typically present a single-crystal structure, for example, body-centered cubic (BCC), face-centered cubic (FCC), or hexagonal-closed packed (HCP). The most prevalently used alloying elements in HEAs are transition metals like Cr, Fe, Co, and Ni, along with Al, Mn, and Ti. Another group consists of refractory metals like Nb, Mo, Ta, and W. These elements can either be incorporated in equiatomic proportions within a single composition or in trace amounts as alloying elements. One of the extensively investigated HEAs is the equiatomic CrMnFeCoNi alloy, known as the Cantor alloy, which solidifies as a single-phase solid solution with an FCC structure. As reported by José M. Torralba et al. [13], about half of the published research on HEAs focuses on the Cantor alloy, highlighting its importance in the field. The elements Fe, Co, and Ni, in equiatomic proportion, serve as FCC stabilizers [14], which help in the formation of a single FCC phase. The concentration of Al significantly influences the microstructure, phase transformation, and performance of HEAs. Approximately 30% of the literature centers on modifications of the Cantor alloy with varying Al additions [13]. Al functions as a strong stabilizer for the BCC phase. For instance, increasing the Al content can transform the crystal structure from FCC to a dual-phase FCC-BCC or entirely BCC [15]. High Al content is generally considered to enhance oxidation resistance [16,17,18] and boost compressive strength, but reduce ductility [19,20]. Nevertheless, excessive Al can cause the formation of brittle intermetallic phases. Ni is an FCC stabilizer in HEAs, and its addition promotes the transformation from BCC to FCC phases [21,22]. Fe is commonly used for its ability to form a stable solid solution with other elements, thereby enhancing the mechanical strength and overall stability of the alloy. Increasing the Fe content in FeCoCrNi alloys raises the BCC phase fraction and improves wear resistance [23]. Cr is a BCC stabilizer and plays a crucial role in HEAs concerning oxidation and wear resistance [17,18,24]. The addition of Co in HEAs further promotes the formation of the FCC phase, which can lead to a decrease in microhardness and strength [25,26]. Mn is often used to improve phase stability and enhance mechanical properties [27,28,29,30]. Ti improves hot corrosion resistance, and when added to Cantor alloys, it promotes the formation of a BCC structure within the FCC solid-solution phase [7,31]. A minor addition of Ti to the CoCrFeNiMn alloy can remarkably increase the thermal stability of the precipitates [32]. However, an excessive amount of Ti can result in the formation of intermetallic compounds and negatively affect ductility. Cu is often added to or substitutes elements in AlCoCrFeNi HEAs because of its favorable mixing enthalpy and weak bonding with other metals, which facilitates the formation of Cu-rich precipitate phases [33]. It is commonly added to HEAs as an FCC-forming element to improve deformability. However, in some alloys, it can lead to the formation of interdendritic segregations, significantly reducing both mechanical properties and corrosion resistance [34]. Si is added to enhance hardness and wear resistance [35,36,37]. While the addition of V leads to the precipitation of a brittle σ phase and causes a significant reduction in ductility [30,38,39], Sm promotes the formation of a refined and uniform microstructure, thereby enhancing corrosion resistance [40]. The careful balance of these elements is vital for optimizing the properties of HEAs. Therefore, the alloy composition is adjusted to modify the performance of HEAs, making them highly promising for specific engineering applications.

### 1.2. Additive Manufacturing Technologies

Additive manufacturing (AM), also known as 3D printing, is a process of joining materials to make parts using 3D model data. This is typically accomplished layer by layer, differentiating it from subtractive manufacturing and formative manufacturing techniques [41]. It serves as a revolutionary manufacturing approach that constructs complex 3D objects layer by layer. Over the past few decades, this technology has introduced remarkable transformations to various industries.

The history of AM can be traced back to about 160 years ago. It is believed that AM originated from the combination of photosculpture, which evolved in the nineteenth century for the construction of sculptures, and topography, which developed in the twentieth century for the creation of topographical maps [42]. Photosculpture techniques placed a greater emphasis on replicating subjects, such as a person, an animal, or an object, to the best possible extent. During the nineteenth century, photosculpture aimed to create precise 3D replicas of any given object. In 1860, the Frenchman François Willème devised a successful technique involving simultaneous photography by 24 cameras [43]. Subsequently, Baese [44], Monteah [45], and Morioka [46,47] made improvements and advancements to this technology. The topography techniques, on the other hand, were more concerned with the accurate depiction of contour data and topological details, which were then utilized in the preparation of maps. As early as 1890, Blanther put forward a layered method for crafting molds for topographical relief maps [48]. Subsequent improvements and developments were made by Perera [49], Zang [50], Gaskin [51], Matsubara [52], DiMatteo [53], and Nakagawa [54,55,56], enabling the use of this method for manufacturing surfaces and tools that are difficult to fabricate by standard machining operations. The integration of photosculpture and topography laid the foundation for the modern era of AM.

Following the middle of the twentieth century, the majority of innovations within AM were associated with the utilization of novel materials and manufacturing approaches. By the 1970s, distinct material-based themes within AM practices had become evident, which eventually evolved into diverse categories of AM technologies, such as powder bed fusion (PBF), sheet lamination (SHL), and vat photopolymerization (VPP). The 1960s and 1970s witnessed the validation of the first modern AM processes, with photopolymerization technology being a notable example in the late 1960s. In 1968, Swainson [57] put forward a process for directly fabricating a plastic pattern through the selective (3D) polymerization of a photosensitive polymer at the intersection of two laser beams, with parallel work conducted at Battelle Laboratories. In 1971, Ciraud [58] proposed a powder-based process that exhibited features of modern, direct-deposition, solid freeform fabrication (SFF) techniques. Housholder [59] provided the earliest description of a powder laser sintering process in 1979. In 1981, Hideo Kodama [60] published a rapid prototyping system based on VPP, while Herbert [61] undertook a similar endeavor at 3M. Nevertheless, during that period, AM technology was still in its infancy, with scarcely any commercial market and minimal investment in research and development. The 1980s and early 1990s witnessed a significant upsurge in the number of AM-related patents and academic publications, accompanied by the emergence of numerous innovative AM technologies. Notably, there was the 3D printing technology (3DP) developed by the Massachusetts Institute of Technology in 1989 and the laser beam melting process in the 1990s. During the same period, several AM technologies achieved commercial success, including stereolithography (SL), fused deposition modeling (FDM), and selective laser sintering (SLS), thereby firmly establishing the foundation of the AM industry as it exists today.

At present, a diverse range of principal technologies have been established, namely SL, PBF, material extrusion (ME), binder jetting (BJT), VPP, SHL, direct energy deposition (DED), direct ink writing (DIW), and additive friction stir deposition (AFSD). Each of these AM technologies possesses its unique working principles and characteristics and is applicable to different application contexts, with the potential for further categorization. For instance, SL encompasses stereolithography apparatus (SLA), which utilizes a laser to carry out the selective photopolymerization of liquid photopolymer resin layer by layer, and digital light processing (DLP), which employs a digital projector to cure an entire layer of resin simultaneously. PBF technology comprises various methods, including selective laser melting (SLM), selective electron beam melting (SEBM), and SLS. SLM is a process that utilizes a high-power laser to melt metal or alloy powders within a powder bed to fabricate complex metal components. SEBM, on the other hand, employs an electron beam for melting, operating under high vacuum conditions. Lastly, SLS sinters powders below the melting point, fusing the particles together. ME technology is mainly exemplified by FDM, which extrudes a thermoplastic filament through a heated nozzle, depositing it layer by layer. BJT technology entails the selective deposition of a liquid binder onto a powder bed to bind particles together. VPP technology involves curing liquid resin through the use of various light sources. SHL technology, including laminated object manufacturing (LOM), involves the cutting and layering of sheets of material. DED technology constructs components by melting and depositing material, employing energy sources such as laser, electron beam, or arc melting. DIW technology extrudes viscous inks or pastes through a nozzle in a controlled pattern. Lastly, AFSD is a distinctive process that combines the principles of friction stir welding with AM. It utilizes a rotating tool to generate frictional heat, which softens the material and allows for the deposition and consolidation of additional material in a layer-by-layer manner, facilitating the production of complex metal components.

In the realm of AM, a wide variety of materials is employed, chosen for their unique properties and their compatibility with specific AM technologies. SL, which includes SLA and DLP, mainly makes use of photopolymer resins. These resins can be customized to display the desired mechanical properties, transparency, and heat resistance. PBF techniques exhibit material preferences that match their process requirements. SLM and SEBM are primarily utilized for metals and alloys, including Ti alloys, Al alloys, and stainless steels; SLS has the ability to process a range of materials, including nylon, polystyrene, and some metal powders. ME technology, represented by FDM, predominantly utilizes thermoplastic filaments such as polylactic acid (PLA), acrylonitrile butadiene styrene (ABS), polyethylene terephthalate (PET), and polycarbonate (PC). BJT shows versatility in material application, covering metals like iron, stainless steel, and Ti, as well as ceramics and some polymers. VPP mainly employs photopolymer resins similar to those used in SL. SHL, like LOM, and uses materials like paper, plastic sheets, and metal foils. DED is mainly for metals and alloys such as Ti, Ni-based superalloys, and some steels. DIW is capable of handling a diverse range of materials, including ceramic pastes, polymer gels, and metal-based inks with adjustable viscosity. AFSD is primarily utilized for metals and alloys, such as Al alloys, Mg alloys, and some Cu alloys, to fabricate components with fine-grained microstructures.

The structures fabricated through AM can exhibit a high level of complexity and diversity. A prominent illustration of this is metal lattice structures, which are constructed from nodes that are interconnected by rods between nodes, expanding in accordance with a defined spatial periodicity [62]. Metal lattice structures have excellent mechanical properties such as being ultra-lightweight and having high porosity, high specific strength, high specific stiffness, high strength and toughness, and high energy absorption, as well as special properties such as sound absorption, shock absorption, heat dissipation, electromagnetic shielding, and excellent permeability [63]. They are multifunctional engineering structural materials with excellent performance and have very promising application prospects in various industries such as aerospace, national defense and military, transportation, and energy. Moreover, AM allows for the production of large thin-walled structures, intricate curved surface structures, as well as integrated structures. Thin-walled structures are widely found in components like aeroengine blades, casings, and combustion chambers. Complex curved surfaces are commonly used in aircraft, ships, and automobiles to meet fluid mechanics requirements. Integrated structures can avoid the need for connecting structures such as flanges and welds when multiple parts are combined.

The objective of this review is to comprehensively summarize the recent advancements in the production of HEAs using AM. It also aims to analyze the advantages and challenges associated with various AM processes, and to offer perspectives and guidelines for future research in this field.

## 2. Additive Manufacturing of HEAs

In recent years, AM has emerged as an effective technique for fabricating metallic materials. This technology enables the fabrication of intricate components based on 3D computer-aided design (CAD) models. The AM technique has increasingly gained prominence in materials science and is widely utilized in industry for manufacturing products with complex geometries. To date, a variety of AM-based methods have been developed and are now commonly employed in the manufacturing processes of various engineering materials, including Ti alloys [64], Al alloys [65], steels [66], Mg alloys [67], Co alloys [68], and composites [69].

In general, HEAs are usually produced through conventional casting and powder metallurgical methods. However, these techniques face challenges in terms of manufacturing components with complex geometries and achieving an ultrafine-grained structure. Moreover, components produced using these traditional methods are susceptible to metallurgical defects owing to their inherently low cooling rates. Currently, the predominant AM techniques utilized for HEAs include SLM [15,70,71,72,73,74,75,76,77,78,79,80,81,82,83,84,85,86,87,88,89,90,91,92,93,94,95,96,97,98,99,100,101,102,103,104,105,106,107,108,109,110,111,112,113,114,115,116,117,118,119,120,121,122,123,124,125,126,127,128,129,130,131,132,133,134,135,136,137,138,139,140,141,142,143,144], SLS [145], SEBM [130,146,147,148,149,150,151,152], DED [75,153,154,155,156,157,158,159,160,161,162,163,164,165,166,167,168,169,170,171,172,173,174,175,176,177,178,179,180,181,182,183,184,185,186,187,188,189,190,191,192,193,194,195,196,197,198], BJT [199,200,201], DIW [202,203], and AFSD [204,205]. The investigation of microstructural evolution, property enhancement, and defect control in HEAs manufactured through these AM methods is of utmost importance and has attracted considerable attention within the academic community.

### 2.1. Selective Laser Melting (SLM)

**Figure 1:**
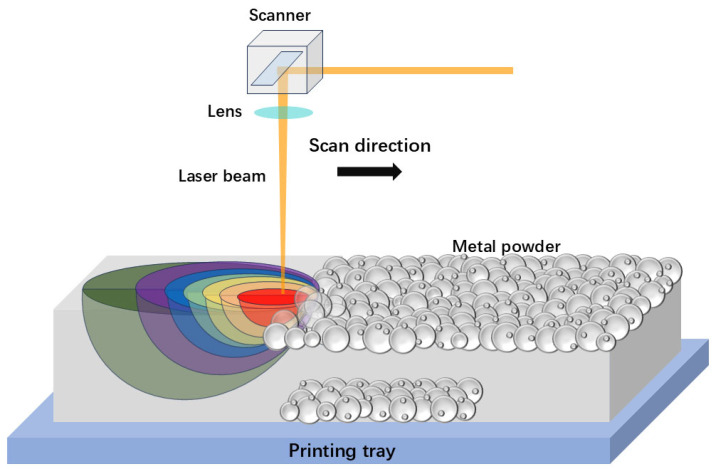
Schematic of typical SLM process.

Figure 1 illustrates the schematic of SLM. SLM, also known as laser powder bed fusion (LPBF), is an advanced AM technique that employs a high-powered laser to selectively melt and solidify specific areas of metallic powders layer by layer, in line with the cross-sectional data of the 3D CAD model. This layer-by-layer method makes it possible to fabricate intricate geometries that are usually impossible or extremely challenging to create using traditional manufacturing approaches. In comparison with SEBM and DED, SLM has several unique advantages. It uses a lower laser power and a finer laser beam with high beam quality. This facilitates rapid heating and cooling rates, which promote the formation of non-equilibrium phases and fine-grained microstructures with innovative properties. Through adjustments in processing parameters such as laser traverse speed, power, beam size, precursor composition, and feed rate, a wide range of microstructures and properties can be tailored using SLM.

Currently, SLM is the most extensively researched AM technique for HEAs. The Cantor alloy (i.e., CoCrFeMnNi) and its derivatives (CoCrFeNi, AlCoCrFeNi, et al.) produced by SLM have been thoroughly investigated.

To the best of my knowledge, Y. Brif et al. [70] conducted the pioneering study on applying SLM to HEAs. Their work demonstrated that a homogeneous single-phase FCC solid solution is obtainable in equiatomic FeCoCrNi HEAs across a range of processing parameters using beam traverse rates of 0.33, 0.36, 0.38, 0.44, 0.48, and 0.5 m s^−1^, and no phase transformations take place up to the melting point of the alloy (1414 °C). The SLM-processed FeCoCrNi alloy, which is characterized by a typical single-FCC structure [71,72,75], exhibits columnar grains that are elongated along the build direction [71,72]. The microstructure has a duplex composition consisting of both elongated phases and nearly equiaxed phases. The elongated phases, whose length is several tens of micrometers, are related to the diameter of the laser spot. The nearly equiaxed microstructures can be found between neighboring tracks, resulting from the overlap of adjacent laser tracks that leads to the remelting and recrystallization of the as-built phase [71,72,73]. Large grain sizes induce severe residual stress, leading to intergranular hot cracks, which adversely affect the printability [74]. By introducing 1.8 at.% N into the alloy, a remarkable enhancement in both strength and ductility is achieved compared to the non-doped alloy [75]. The N-doped HEA exhibits an exceptional combination of tensile strength and elongation, surpassing many other single-phase FCC HEAs. In the study by K. Zhou et al. [135], the SLM-processed Ni_2.1_CoCrFeNb_0.2_ HEA exhibited a random distribution of the γ″ phase within the FCC matrix after aging treatment. This γ″ phase was identified as a typical D0_22_ ordered structure, characterized by the crystallographic relationships [001]_γ″_//<001>_γ_ and {100}_γ″_//{100}_γ_. As the aging time increased, the volume fraction of the γ″ phase also rose, which brought about a significant enhancement in tensile strength, thus emphasizing its considerable strengthening effect in this newly developed HEA.

The Cantor alloy, namely the equiatomic CrMnFeCoNi alloy, stands as one of the HEAs that has received the most extensive investigation. In the SLM process, it has been found that the CoCrFeMnNi HEA can be composed of either a single BCC [80,81,82] or a single FCC phase [83]. The CrMnFeCoNi HEA fabricated through SLM reveals a complex hierarchical microstructure along the build direction. This microstructure encompasses the generation of nanocrystalline grains, elemental segregation, precipitation phenomena, cellular dislocation structures, deformation twinning, and phase transformations triggered by deformation [76,77].

The rapid cooling and repeated metal deposition within the SLM process give rise to epitaxial growth and competitive grain growth, which exert a substantial influence on the microstructural development and mechanical properties of the alloy [78]. The rapid cooling leads to the formation of extremely fine, rod-like cells. Owing to the presence of existing cells (or grains) beneath the layers, cubic crystals are witnessed to grow epitaxially from these pre-existing cells (or grains) during the rapid cooling in the powder-bed laser fusion of cubic alloys. It has been identified that the preferred growth direction of the cells is the maximum heat flux direction [79]. The heat flux in the new meltpool serves as a decisive factor that governs the direction of crystal growth. In the case where the direction of existing cells coincides with the direction of heat flux in a new meltpool, the cells continue to grow epitaxially without any alteration in their growth direction. Conversely, a disruption in the growth direction occurs. When the direction of existing cells is perpendicular to the heat flux in the new meltpool, the cells are observed to branch out from the existing ones sideways, resulting in epitaxial growth with a 90-degree change in the growth direction.

The CrMnFeCoNi sample processed by SLM demonstrated a homogeneous chemical distribution [80,81,82]. Nevertheless, the study by M. Jin et al. uncovered segregation patterns during the solidification of CrMnFeCoNi. Specifically, Cr, Co, and Fe accumulate in the cores of solidification cells, while Mn and Ni have a tendency to segregate towards the cell boundaries [84]. N. Choi et al. [85] probed into the non-equilibrium state of general high-angle grain boundaries (HAGBs) in the AM-processed CoCrFeMnNi HEA. Their findings imply that these grain boundaries exhibit a non-equilibrium condition similar to that found in severely plastically deformed materials.

The addition of Mn results in the formation of oxides in the CoCrFeMnNi HEA [76,81,82,83,84,86]. The oxide particles in the HEA are identified to be Mn_2_O_3_ and MnO [81,82]. The Mn_2_O_3_ phase most likely resulted from the remelting of the oxide surface of elemental Mn powder, while the MnO particles should be due to the in situ oxidation reaction between Mn and O during the AM process. Oxides not only increase strength but also enhance creep resistance [83]. The enhanced strength is primarily attributed to dislocation hardening and nanoscale oxides [77,83,87]. R. Li et al. [88] reported that the SLM-processed CoCrFeMnNi HEA revealed unique microstructural features, including nanotwins and tetragonal σ phase, which significantly enhanced the mechanical properties of the alloy through grain refinement and synergistic strengthening effects.

P. Litwa et al. [89] investigated the machining characteristics of SLM-manufactured CrMnFeCoNi HEA and demonstrated superior machinability of the CrMnFeCoNi alloy over AISI 304L, attributed to its plastic behavior characterized by substantial ductility and adequate strength. J. Guo et al. [90] also investigated the machinability of SLM-processed CoCrFeMnNi HEA, evaluating the effects of various machining processes on the surface and subsurface quality. Their study explored correlations among microhardness, residual stress, and subsurface deformation in the HEA.

Al is an important additive element in HEAs, and the AlCoCrFeNi HEAs is one of the most studied HEA systems [15,97,98,99,100,101,102,103,104,105,106]. HEAs within this system consist of solid solution phases, e.g., BCC, FCC, or both, depending on the Al concentration or Al/Ni ratio [15,99,107,108]. At low Al concentrations, AlxCoCrFeNi forms a single disordered FCC phase, as observed in 0Al and 0.1Al samples. As the Al content increases, a mixture of BCC (disordered A2 or ordered B2) and FCC phases emerges. When Al exceeds a threshold (x ≈ 0.9), the alloy becomes a single BCC phase. However, Y. Liao et al. [133] also found only a single BCC phase in the SLM-processed Al_0.5_FeCrNi_2.5_V_0.2_ HEA. F. Peyrouzet et al. [109] investigated the SLM-processed Al_0.3_CoCrFeNi HEA, which demonstrated excellent printability and exhibited a single-phase disordered FCC solid solution with a <110> fiber texture aligned along the build direction. K. Sun et al. [111] found that the microstructure of SLM-processed Al_0.5_CoCrFeNi HEA revealed a composition comprising FCC and BCC phases, without complex intermetallic compounds. Z. Sun et al. [112] designed Al_0.1_CoCrFeNi, Al_0.5_CoCrFeNi, and Al_1.0_CoCrFeNi HEAs to study the effects of Al on phase evolution. During solidification, Al partitions into interdendritic and grain boundary regions, and after B2 solidifies, the remaining liquid forms an FCC matrix that transforms to B2 through solid-state phase transformation. Eventually, B2 and BCC mixtures result from spinodal decomposition. The introduction of Al, particularly in Al_0.5_CoCrFeNi, significantly reduced hot crack density in hot-cracking-susceptible CoCrFeNi HEA. Y. Su et al. [113] investigated the impact of Al content on the microstructure and cracking behaviors of SLM-produced AlxCrCuFeNi_2_ HEAs (x = 0, 0.5, 0.75, 1.0). They observed a transition from BCC to FCC plus BCC/B2 structures with increasing Al, accompanied by a shift from columnar to equiaxed grain morphology. Alloys with Al0.75 and Al1.0 displayed a eutectic-like microstructure of lamellar/cellular FCC matrix and interdendritic B2 matrix with spherical BCC nano-precipitates. The FCC dendrites and BCC nano-precipitates were enriched in Fe and Cr, while the B2 matrix was enriched in Al and Ni. A phase transformation from BCC to FCC phases in SLM-processed Al_0.5_FeCoCrNi HEA during processing was also observed by P.F. Zhou et al. [110]. The cracking mechanisms shifted from intergranular hot cracking in coarse FCC grains to transgranular cold cracking in brittle BCC grains due to severe residual stress, correlating with the phase shift. The equiaxed structure in Al_0.75_ reduced hot cracking, and the eutectic-like microstructure effectively suppressed cold cracking, showcasing their combined effect in crack prevention. However, D. Karlsson et al. [114] found that, despite exploring a wide process parameter window, crack-free SLM-produced AlCoCrFeNi HEAs were unattainable due to inherent stresses during the build process.

H. Yao et al. [142] studied the SLM-processed AlCrFeNiV HEA and found a complex microstructure with columnar grains, sub-grains, L1_2_ nano phase, and dislocations. The columnar grains, ranging from tens of microns to 200 μm, grew along the temperature gradient. Rapid cooling and non-equilibrium solidification during SLM caused sub-grains to form within each columnar grain, leading to a heterogeneous distribution of dislocations and the L1_2_ nano phase. The SLM-processed AlCrFeNiV HEA demonstrated high strength and notable ductility, primarily attributed to the sub-grains that enhance dislocation hardening and the presence of the L1_2_ nano phase within the sub-grains. D. Karlsson et al. [114] found that AlCoCrFeNi exhibits B2 ($Pm\bar{3}m$) and BCC ($Im\bar{3}m$) phases. Their comparison revealed that induction-melted samples had large, randomly oriented grains, whereas SLM-produced samples featured smaller dendritic grains with nm-scale chemical fluctuations. Annealing increased chemical heterogeneity, leading to the formation of Cr-rich and Cr-poor regions. The SLM-printed AlCoCrFeNi (Al = 10 at%) HEA contained dual phases of disordered BCC (A2) and ordered BCC (B2), differing from conventional cast and deformed samples, which had A2 and FCC phases due to the rapid cooling rate of SLM [115]. Notably, Fe-Cr precipitates were present in SLM samples without additional heat treatment. The microstructure showed epitaxial growth of columnar A2 grains averaging 1.5 μm, with B2 phases between them [115]. D. Vogiatzief et al. [116] investigated AlCrFe_2_Ni_2_ HEA, which features FCC and BCC phases, where the BCC phase undergoes spinodal decomposition during heat treatment. S. Sarkar et al. [132] investigated SLM-processed AlCoFeNiTiV_0.9_Sm_0.1_ and AlCoFeNiV_0.9_Sm_0.1_ HEAs and found only a single BCC phase in both alloys. P.K. Sarswat et al. [143] investigated SLM-fabricated AlCoFeNiV_0.9_Sm_0.1_, AlCoFeNiSm_0.1_TiV_0.9_, AlCoFeNiSm_0.05_TiV_0.95_Zr, and a bulk metallic glass AlCoFeNiTiVZr, discovering that most of these alloys exhibited a single-phase FCC structure. Moreover, first principles calculations and experimental evaluations demonstrated that these alloys possess exceptional mechanical strength and corrosion resistance properties.

As a common additive in steel that induces significant changes in the mechanical properties of Ni-based and Co-based superalloys, C is also incorporated into HEAs to enhance their strength [100,206,207,208,209,210]. The addition of C to HEAs typically results in the formation of precipitate carbides, leading to significant precipitation strengthening [210,211,212,213]. However, carbides precipitated in HEAs produced through traditional processing methods are typically in the submicron to microscale range, which can notably reduce ductility [210,211,212,213]. Employing SLM, the addition of C in HEAs results in the formation of nano-sized carbides [117,118,119,120,121,122]. W. Wu et al. [117] investigated nanosized precipitates and dislocation networks in an SLM-fabricated CoCrFeNiC_0.05_ HEA, revealing cellular and columnar subgrain structures with Cr-segregated dislocation networks and nanosized M_23_C_6_-type carbides at grain boundaries. The orientation of M_23_C_6_ to the matrix was identified as (111)_M23C6_//(111)_matrix_; [011]_M23C6_//[011]_matrix_. Enhanced strength resulted from interstitial solid solution, dislocation, and precipitation strengthening. In contrast, R. Zhou et al. [118] found a homogeneous carbon distribution in FeCoCrNiC_0.05_ HEA without carbides. Y.K. Kim et al. [119] demonstrated that the higher C content in CoCrFeNiCx HEAs improved tensile strengths and elongation due to precipitation hardening, easy formation of deformation twins, and higher back stresses, along with reduced pore and micro-crack densities. Z. Li et al. [123] reported that high C content in CoCrFeMnNi HEAs reduces mechanical twinning activity due to increased stacking fault energy (SFE), with twins only appearing at high strain levels. Conversely, J.M. Park et al. [120] observed increased twinning activity in tensile-deformed samples with higher C content. In their other work [121], superior tensile properties of the SLM-fabricated CoCrFeMnNiC_1_ HEA were achieved by interstitial solid solution and precipitation hardening. J. G. Kim et al. [122] highlighted solute heterogeneities in C-doped CoCrFeMnNi HEAs, noting that interstitial elements in C-CoCrFeMnNi powders interacted with Mn and Cr during AM, forming nano-sized precipitates and inducing dislocation cell networks. These heterogeneities create plastic strain incompatibility, leading to additional geometrically necessary dislocations (GNDs) that relieve it. The accumulated GNDs provide high back stress, resulting in significant mechanical improvements. Z. G. Zhu et al. [134] fabricated an interstitial solute-strengthened SLM-processed C-doped Fe_49.5_Mn_30_Co_10_Cr_10_C_0.5_ (at.%) HEA in which all elements among the cells were uniformly distributed without apparent segregation.

Si has been added to the FeCoCrNi HEA system to maintain its single-phase FCC solid-solution structure without the formation of secondary or precipitate phases [124]. A single FCC solid solution could only exist in the FeCoCrNi system at a Si content below 5.88% [124]. D. Lin et al. [124] synthesized a FeCoCrNi HEA with 1.5 at.% Si using SLM, revealing columnar grains of a single-phase FCC solid solution without the formation of secondary phases. Its strong tensile properties were attributed to solid solution and dislocation strengthening. P. Agrawal et al. [125] investigated a metastable SLM-fabricated Fe_40_Mn_20_Co_20_Cr_15_Si_5_ HEA. They observed that the as-printed HEA exhibited enhanced strength attributed to its high work hardenability while maintaining significant uniform ductility through a combination of transformation and twinning-induced plasticity during deformation. Moreover, the alloy demonstrated excellent printability with minimal void content (~0.1%). L. Guo et al. [126] studied the effects of N and Si on the grain size distribution, elemental segregations, mechanical properties, and hot cracking behaviors in an SLM-fabricated FeCoCrNiMn HEA. Their findings indicated that N and Si promoted preferential segregations of Mn and Ni, thereby facilitating intergranular hot crack initiation and propagation. The formation of a hierarchical microstructure involving elemental segregations, dislocation cells, nanotwins, fine precipitates, and multimodal grain structure enhanced mechanical properties while underscoring the need to optimize processing parameters in order to prevent dendritic growth and segregation.

Cu is frequently added to or replaces elements in Al-Co-(Cr)-Fe-Ni HEAs due to its positive mixing enthalpy with other elements, facilitating the formation of Cu-rich precipitate phases [33,214,215]. Adding Cu can easily induce severe interdendritic segregation and impair the mechanical properties [216]. S. Luo et al. [128] employed SLM to fabricate an equiatomic AlCrCuFeNi HEA and revealed unique microstructural features such as fine columnar grains with a <100> preferred orientation and nano-scale Cu-rich phases precipitating along grain boundaries, contributing to enhanced mechanical properties. In their other works [217,218], they explored the SLM fabrication of dual-phase HEAs derived from a BCC AlCrCuFeNix (2.0 ≤ x ≤ 3.0) HEA. They found that the B2 lamellae are abundant in nano-precipitates, whereas the FCC lamellae show no secondary phases. B2 lamellae are enriched in Al and Cu, while FCC lamellae are rich in Cr and Fe. Additionally, there is a slight increase in the concentrations of Al, Cu, and Ni at the FCC/B2 phase boundaries. Zhang et al. [127] found that SLM-processed AlCoCuFeNi HEA exhibited a singular ordered BCC (B2) solid solution phase and a refined columnar substructure oriented strongly along the layer build-up direction. Subsequent heat treatment at 900 °C and 1000 °C induced the precipitation of a Cu-rich BCC phase from the metastable BCC (B2) matrix, resulting in a dual-phase microstructure in the treated alloys. Y. Wang et al. [129] noted that the SLM-produced AlCoCrCuFeNi HEA exhibited a significant presence of both FCC and BCC phases, with crack formation attributed to HAGBs, Cu element segregation, and phase misfit.

In HEAs, Ti can be added as equiatomic proportions in one composition or as the minor alloying element. The addition of Ti is known to enhance the mechanical properties and thermal stability of the CoCrFeNiMn HEA through stabilizing precipitates [32]. T. Fujieda et al. [130] discovered that the SLM-processed CoCrFeNiTi-based HEA exhibited a fine and uniform microstructure without visible segregation. After the solution treatment, they noticed an increase in the volume fraction of extremely fine-ordered particles with a diameter in tens of nanometers, containing Ni and Ti. X. Yang et al. [137] developed a novel alloy, Ni6Cr4WFe9Ti, which has a fine-grained structure and mainly consists of the γ phase. In this alloy, Ti and W were preferentially incorporated into the lattice Ni (100) site of the γ phase, acting as donors. Z. Gu et al. [136] conducted research on a new HEA, CoCr_2.5_FeNi_2_TiW_0.5_, using SLM technology for AM. They found that, under Ar protection, the alloy formed a single-phase solid solution, whereas under N_2_, a second-phase TiN appeared, leading to a BCC matrix with TiN precipitates. The samples protected by N_2_ exhibited higher average microhardness values and improved mechanical properties compared to those protected by Ar. W.C. Lin et al. [131] found that SLM-processed Al_0.2_Co_1.5_CrFeNi_1.5_Ti_0.3_ HEA samples aged directly at 750 °C for 50 h exhibited a microstructure with dispersed L1_2_ particles and newly formed L2_1_ phases at subgrain boundaries, along with nano oxides and dislocations in the FCC matrix. A high Ti content may stabilize the L2_1_ phase over the B2 phase. The L1_2_ phase features an ordered FCC structure with Ni, Co, and Fe at the face-centered lattice points and Al and Ti at the corners. In contrast, the L2_1_ phase possesses an ordered BCC structure, with Ni at the corners and Al and Ti at the center lattice points.

NbMoTaW is a widely studied BCC equiatomic refractory HEA. H. Zhang et al. [139] utilized SLM to produce an NbMoTaW refractory HEA, achieving a single BCC solid solution. Their analysis revealed a slight negative deviation in the molar ratio of elements with lower melting points and lower density within the alloy. The resultant alloy exhibited superior microstructural integrity, microhardness, and corrosion resistance compared to traditional superalloys, suggesting promising applications in the aerospace and energy sectors. Furthermore, they explored the thermal–mechanical behavior of the SLM-fabricated WTaMoNb HEA, revealing significant warping and cracking tendencies attributed to uneven temperature distribution, which were successfully mitigated through improved process adjustments informed by thermal–mechanical simulations [138]. The NbMoTa HEA showed severe cracking defects during the SLM process, where the addition of Ti reduced the crack size and the addition of Ni eliminated the microcracks. H. Zhang et al. [140] also developed a NbMoTaTi0.5Ni0.5 HEA with notable room-temperature compressive strength (2297 MPa) and high-temperature (1000 °C) compressive strength (651 MPa) using SLM, demonstrating enhanced formability through the suppression of crack formation and microcrack transformation at grain boundaries with low SFE. The simultaneous addition of both Ni and Ti suppressed the formation of cracks and defects in an SLM-prepared NbMoTaTi_0.5_Ni_0.5_ alloy as well as ensured high room-temperature and high-temperature compressive strength.

T. Ishimoto et al. [141] developed pre-alloyed Ti_1.4_Nb_0.6_Ta_0.6_Zr_1.4_Mo_0.6_ HEA powders and fabricated SLM-built components with low porosity, customizable shapes, high yield stress, and excellent biocompatibility. Addressing challenges such as poor shape customizability and severe elemental segregation inherent in HEAs, the study employed an extremely high cooling rate (~10^7^ K/s) during SLM.

There are also some studies on the preparation of HEA composites using SLM. Most of them focus on reinforcing CoCrFeNiMn HEAs with TiN_p_ [93,94,95]. The as-printed composites exhibited refinement of the HEA matrix grain structure as well as improvements in mechanical properties due to the addition of TiN_p_. The TiN_p_ significantly improved the microstructure of the HEA matrix, resulting in the formation of refined and isotropic grains in contrast to the anisotropic coarse-grained structure that was observed in TiN_p_-free HEA prints [93,94]. The TiN_p_ exhibited uniform distribution within the HEA matrix, contributing to strengthening through pinning effects. The introduction of TiN_p_ during SLM acted as numerous nanoscale nucleation sites, promoting the formation of nearly equiaxed and ultrafine grains in the HEA matrix, thereby enhancing its mechanical properties. N. Li et al. [96] utilized SLM to fabricate composites composed of Fe_20_Co_20_Cr_20_Ni_20_Mn_20_ (at.%) HEA and Fe_43.7_Co_7.3_Cr_14.7_Mo_12.6_C_15.5_B_4.3_Y_1.9_ (at.%) amorphous, which combined high strength and toughness. The intriguing finding is that two different high entropy phases in addition to the amorphous phase were detected at the HEA and metallic glass interfacial region. With increasing the fraction of Fe-based metallic glass, the composite exhibited enhanced strength and excellent fracture toughness. Y. Peng et al. [144] investigated the interface behavior in a Ti-coated diamond and FeCoCrNi HEA composite produced via SLM, focusing on the dual influence of diffusion barrier and interfacial strengthening. First-principles simulations and experimental validation revealed that the formation of TiC at the interface acted effectively as a diffusion barrier, mitigating the sp^2^ hybridization of diamond and thereby enhancing the bonding strength across the interface with the HEA matrix. The TiC layer prevented direct contact between diamond particles and transition metal elements in the HEA, thereby inhibiting diamond graphitization and further enhancing interfacial bonding strength. Additionally, Ti from the coating diffused into the HEA matrix, inducing lattice distortion that contributed to substitutional solution strengthening, thereby improving the retention of diamond abrasives.

### 2.2. Selective Laser Sintering (SLS)

SLS is similar to SLM, but it uses a laser to sinter the powder bed rather than melting it. This allows for the production of less dense materials for specialized applications. In the realm of HEAs, the existing literature concerning SLS is rather scarce. Only one paper has been found that pertains to HEAs fabricated by the SLS technique. X. Yan et al. [145] prepared the Ni_30_Cr_25_Al_15_Co_15_Mo_5_Ti_5_Y_5_ HEA coatings on P355GH via SLS under different working modes of pulsed/CW lasers in the atmosphere to improve the surface performance of pressurized light-transmission pipes. The effect of laser-induced plasma on the SLS process was investigated by analyzing the micromechanics, friction, and wear behavior of the HEA coatings.

### 2.3. Selective Electron Beam Melting (SEBM)

**Figure 2:**
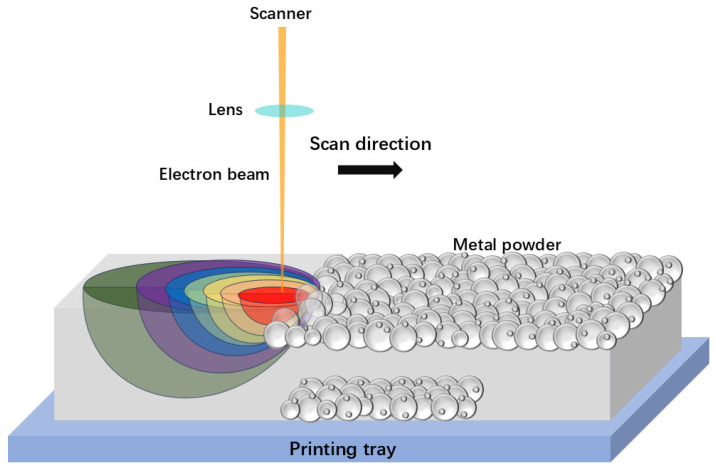
Schematic of typical SEBM process.

SEBM, also known as electron powder bed fusion (EPBF), is an alternative AM technique based on the powder bed system that utilizes an electron beam instead of a laser beam, as illustrated in Figure 2. The focused electron beam sequentially melts the points built in the slice data obtained from the 3D CAD file. As a result, this fundamental difference requires SEBM to operate under a high vacuum system (above 10^−4^ mbar) to ensure a high-quality electron beam and a contamination-free processing environment. Furthermore, compared to SLM, SEBM employs a larger beam size and a thicker layer thickness ranging from 50 to 200 µm, necessitating the use of spherical metallic powders of a larger size for printing. The cooling rate in SEBM is usually two orders of magnitude lower than that in SLM, leading to slightly poorer dimensional accuracy and surface quality of the built part compared to the SLM-produced part. In particular, the SEBM process begins with a high preheating temperature (up to 1100 °C) of the powder bed through a defocused electron beam, high beam current, and speed values. This step can slightly sinter the powders, resulting in a reduction in the temperature gradient and cooling rate (10^3^–10^5^ K s^−1^) in SEBM compared to SLM. Consequently, the hot cracking and construction failure during the printing process caused by thermal stress can be avoided to some extent.

The microstructure of specimens produced via SEBM showed fine columnar grains with the texture of {100} along the building direction [146,147,148]. H. Shiratori et al. [149] investigated the microstructure of SEBM-fabricated equiatomic AlCoCrFeNi HEAs, revealing a predominant nano-lamellar mixture of disordered BCC and ordered BCC (B2) phases, with precipitation of BCC phases at grain boundaries influenced by the unique preheating process of SEBM. K. Kuwabara et al. [146] investigated the SEBM fabrication of equimolar AlCoCrFeNi HEAs and discovered that the top part the SEBM specimen featured BCC-based structures, whereas the bottom part contained both BCC and FCC structures. The precipitates exhibited the same grain orientation as the matrix, suggesting that the phase separation was an order–disorder phase transformation. The areal ratio of the FCC formed in grain boundaries increased from 7% in the top part to 19% in the bottom part. T. Fujieda et al. [147] found that the casting AlCoCrFeNi specimen and raw powders had BCC single-phase structures, whereas the SEBM AlCoCrFeNi specimen had both BCC and FCC structures. This is assumed to be attributed to the preheating process, as both BCC and FCC phases are thermodynamically stable at the preheating temperature. P. Wang et al. [148] found that SEBM-built CoCrFeNiMn HEA components exhibited a hierarchical microstructure, featuring long columnar grains and intragranular cellular structures (dendrites) aligned along the build direction. V.V. Popov et al. [150] found that a notable Al evaporation occurred through the SEBM of Al_0.5_CrMoNbTa_0.5_ HEAs. The microstructure of these SEBM-processed Al_0.5_CrMoNbTa_0.5_ HEAs was composed of two solid solutions: matrix phase corresponding to TaMoNb-based solid solution with low (1.4 at. %) Al content, and second (minor) phase corresponding to (TaMoNbCr)Al solid solution with relatively high (~11.8 at. %) Al content [150].

The microstructure developed during the SEBM process significantly influenced both the mechanical and electrochemical characteristics of the HEAs [130,146,147,151]. The SEBM-produced HEA molds exhibited significantly enhanced mechanical properties compared to conventionally cast counterparts [146,147,151]. This enhancement was attributed to the microstructural refinement [130] and the homogeneous precipitation [151]. Particularly noteworthy was the substantial improvement in ductility, alongside a fracture strength exceeding 1400 MPa, more than six times higher than that of SUS304. Mechanical testing indicated that the deformation mechanism of SEBM-built CoCrFeNiMn HEA primarily involved dislocation activity, with limited contribution from mechanical twinning [148]. Additionally, electrochemical measurements conducted in artificial seawater indicated distinct corrosion behaviors; SEBM AlCoCrFeNi specimens exhibited lower pitting potential (0.112 V vs. Ag/AgCl) compared to cast specimens (0.178 V vs. Ag/AgCl) [146].

As discussed above, PBF methods using a laser or an electron beam as the heat source are often used, and these technologies are capable of creating complex-shaped functional and/or structural components. A notable exception is the work by B. Dong et al. [152], who developed a new powder-bed arc additive manufacturing (PAAM) process incorporating online remelting of deposited material for the fabrication of HEAs. The AlCoCrFeNi_2.1_ HEA was investigated after applying multiple remelting cycles per layer. The results revealed a pseudo-eutectic microstructure comprising predominantly large columnar grains of the FCC phase (~90 wt%) and finer dendritic grains of the BCC phase (~10 wt%). The layer-remelting process significantly reduced the tensile strength and ductility of the alloys due to localized thermally induced plasticity.

### 2.4. Directed Energy Deposition (DED)

Although PBF AM technologies offer advantages such as high geometrical accuracy, they also have drawbacks, including low deposition rates, limited sample sizes constrained by powder bed dimensions, and significant material waste. However, these constraints can be overcome by DED technology.

**Figure 3:**
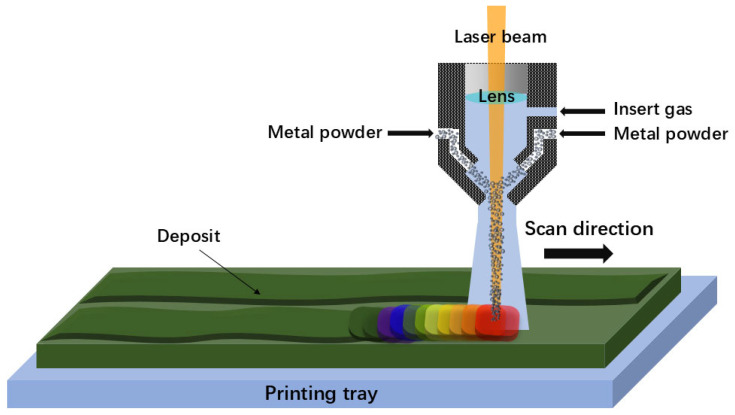
Schematic of typical DED process.

DED is an additive manufacturing process where focused thermal energy is utilized to fuse materials through melting during the deposition process [41]. It is an advanced manufacturing technology developed based on rapid prototyping and laser cladding technology. In the DED process, the metal raw materials are directly melted layer by layer using various energy sources, such as laser beams, electron beams, or arc melting. The feedstock for this process can either be in the form of powder, using blown powder deposition technology, or wire, using arc and laser melting as the heat source. Figure 3 schematically depicts the setup of the DED technology. The powders or wires are transported by the inert gas or wire feed system into an energy source such as a laser beam, melted, and then deposited onto the workpiece to fuse with the previously deposited layer, thereby achieving direct manufacturing and repair of metal parts. By layering the 3D CAD model of a part, the two-dimensional contour information of each layer’s cross-section is obtained, and the processing path is generated. DED can produce 3D products with an ultrafine microstructure and highly complex geometry based on the layer-by-layer incremental shaping and consolidation of the feedstock to a wide range of configurations. Unlike PBF processes that have a constant layer thickness, the layer thickness in DED processing is closely linked to the powder feeding rate and deposition rate. Moreover, DED usually constructs HEA parts at a relatively higher building rate of approximately several tens of cm^3^/min, which is advantageous for the production of larger structures. Notably, DED-processed HEA products can be made through multiple hoppers using several original elemental powders rather than pre-alloyed powders, thus avoiding the time-consuming process of developing HEA powders. This feature endows DED with a great potential for producing large-sized graded HEAs via in situ alloying. Nevertheless, because of the inherent constraints of technical principles, components fabricated by the DED process might show low dimensional accuracy and limited geometric complexity.

In the 1990s, DED technology was independently developed by various international research institutions and assigned various names, including but not limited to: direct energy deposition (DED), laser direct casting (LDC), laser metal deposition (LMD), direct metal deposition (DMD), laser 3D printing, laser consolidation (LC), laser cladding, laser metal forming (LMF), laser rapid forming (LRF), laser 3D printing, laser engineered net shaping (LENS), laser metal deposition (LMD), directed laser fabrication (DLF), direct laser deposition (DLD), laser forming (LF), laser additive manufacturing (LAM), laser-based free entity manufacturing (LBFEM), laser-based free-form fabrication (LBFFF), laser solid forming (LSF), laser forming (LF), wire laser additive manufacturing (WLAM), powder-bed arc additive manufacturing (PAAM) [152], laser-aided additive manufacturing (LAAM) [153], wire arc additive manufacturing (WAAM), and powder plasma arc additive manufacturing (PPAAM). Despite the various names, the fundamental technological principles behind these processes are essentially the same. Among the techniques categorized under the DED category, several have been specifically tailored for the processing of HEAs. These are as follows: DED [154,155,156,157], DMD [158], laser 3D printing [159,160], LENS [161,162,163,164,165,166,167,168,169,170,171], LMD [75,172,173,174,175,176,177,178,179,180,181,182,183,184,185], DLF [186,187,188,189,190], DLD [191,192,193], LAM [194,195], and PPAAM [196].

The microstructure of HEAs can be influenced by factors like laser parameters, scanning speed, and preheating. For instance, the research of I. Kunce et al. [167] and M. Dada et al. [162] demonstrated the impact of these factors on grain size, phase distribution, and hardness. Compared to their traditionally cast counterparts, as-built HEAs showed a heterogeneous grain-size distribution and a high dislocation density, as well as enhanced tensile properties [153,155]. Compared to conventionally cast HEAs, the DED-fabricated HEAs exhibited significantly elevated strength, attributed to refined grains and quantitatively explained by grain boundary strengthening [153]. Moreover, the as-built HEA demonstrated enhanced tensile properties at low temperatures, facilitated by the occurrence of deformation twins and resulting in steady strain-hardening behavior [153]. Preheating plays a critical role in enhancing the mechanical properties of laser AM-processed HEAs by mitigating crack formation induced by thermal stresses [157]. Deformation behavior analysis using in situ high-resolution digital image correlation methods indicated that these differences in mechanical properties were closely associated with the texture developed during deposition [155].

The most extensively studied alloy using the DED method is the CrMnFeCoNi HEA. The majority of studies have reported that DED-processed CrMnFeCoNi HEA exhibits a single-phase FCC structure [167,168,174]. In the study by Z. Tong et al. [195], the DED-produced FeCrCoMnNi HEA maintained a single FCC solid solution after heat treatment. However, X. Gao and Y. Lu [159] found that the DED-fabricated CoCrFeMnNi HEA exhibited a fine BCC phase distributed at the grain boundaries of the FCC matrix. S. Xiang et al. [173] explored the influence of laser power and scanning strategies on microstructural features and tensile properties. Their study shows that changes in these factors significantly influence the transition from columnar to equiaxed microstructures in DED-prepared CrMnFeCoNi HEAs, primarily due to variations in heat flux direction and temperature gradients. They also found that the relative proportions of columnar and equiaxed grains in DED-fabricated CrMnFeCoNi HEAs can be adjusted by modifying the laser power [175]. H. Li et al. [180] used DED to fabricate a single-track CoCrFeMnNi HEA and found that a lower temperature gradient to solidification rate ratio linked to the transition from columnar to equiaxed morphology. H. Li et al. [174] investigated the effects of thermal constraints, thermal cycles, and temperature gradients along the deposition direction on the residual stress distribution in a DED-fabricated CoCrFeMnNi HEA, finding that lower thermal gradients during the process reduced residual stress levels, thereby influencing distortion and the alloy’s functional properties. The DED-fabricated HEAs exhibited a microstructure characterized by directional solidification, transitioning from dendritic columnar grains near meltpool boundaries to equiaxed grains farther away [153,155]. At low scanning speeds, grains tend to be nearly fully equiaxed, while high scanning speeds result in columnar grains [155]. The HEAs with equiaxed grains exhibit higher work hardening rates than their columnar counterparts, with the former exhibiting intergranular microcracks and the latter displaying intragranular microcracks [155]. The DED process, with its small meltpool sizes and rapid cooling rates, induces a significant solute-trapping effect, promoting a more homogeneous distribution of elements compared to conventional casting methods [173]. However, Y. Chew et al. [153] found that the grain boundaries are rich in Ni and Mn, with lower contents of Co, Cr, and Fe. S. Guan et al. [167] investigated solidification conditions, phase formation, microstructural characteristics, and tensile behavior, revealing microstructures that included columnar grains, solidification substructures, and dislocation substructures across multiple length scales. Tensile deformation was predominantly accommodated by dislocation activities and deformation twinning. Z. Qiu et al. [194] discovered that the initial high dislocation density introduced by DED significantly enhances the yield strength (YS) of CrMnFeCoNi HEA, with dislocation motion identified as the predominant deformation mechanism. Moreover, substantial deformation twinning was observed at higher strain levels under cryogenic conditions.

I. Kunce et al. [162] found that DED-fabricated HEA AlCoCrFeNi revealed a BCC-derivative B2-ordered crystal structure. Precipitates within the alloy displayed varied morphologies, from a fine, spherical (diameter < 100 nm) in dendritic regions to spinodal (<100 nm thickness) in interdendritic regions. In the study by R.J. Vikram et al. [164], it was found that the DED-processed AlCoCrFeNi_2.1_ HEA exhibited dendritic and eutectic features, consisting of ordered FCC (L1_2_) and BCC phases. The L1_2_ phase was more prevalent along the build (X) direction, while the BCC phase predominated in the build plane (Z direction). Ni served as the primary base element, with the L1_2_ phase being deficient in Al and the BCC phase deficient in Cr but enriched in Al. The stability of the L1_2_ phase was influenced by the nearly equiatomic distribution of Co, Cr, and Fe. Additionally, the Kurdjumov–Sachs (KS) orientation relationship governed the alignment between the L1_2_ and BCC phases in different build directions. S. Yang et al. [160] investigated the printability and wear characteristics of the AlCrFeCoNi alloy. The microstructure analysis of the printed HEA layer indicated the presence of a sole BCC structure phase. The study highlighted a dendritic–interdendritic microstructure in the printed alloy, demonstrating notable hardness and superior resistance to wear. R. Wang et al. [187] explored the evolution of the microstructure, mechanical properties, and corrosion behavior of AlCoCrFeNi HEA-fabricated DLF. The study investigated both as-deposited samples and those aged at temperatures ranging from 600 °C to 1200 °C for 168 h. Initially, the high cooling rate during DLF promoted the formation of a nearly single B2 solid solution structure, inhibiting FCC phase development. Upon aging, however, intergranular needle-like and plate-like FCC phase precipitates appeared, accompanied by wall-shaped FCC phase precipitates along grain boundaries. This phase evolution led to reduced compressive YS and increased ductility due to the softer nature of the FCC phase compared to the B2 phase. Additionally, the alloy exhibited galvanic corrosion susceptibility, where preferential corrosion of the B2 matrix was observed due to the potential difference between Fe-Cr-rich FCC phases and the Al-Ni-rich B2 matrix. V Ocelík et al. [193] investigated the effects of laser surface processing parameters on the microstructure of AlCoCrFeNi HEAs, highlighting the significant influence of the solidification rate on the phase amounts, chemical composition, and spatial distribution of FCC and BCC phases in these two-phase alloys. S. Guan et al. [166] investigated the AM of the non-equiatomic HEA AlCoCrFeNiTi_0.5_ using LENS. Unlike traditional alloys, which typically exhibit columnar grain structures, the as-deposited AlCoCrFeNiTi_0.5_ specimens displayed a fully equiaxed grain microstructure across a broad range of temperature gradients and solidification velocities. The primary microstructural features consisted of B2-structured proeutectic dendrites surrounded by lamellar or rod-like B2/A2 eutectic structures. The fragmented nature of the proeutectic B2-structured dendrites observed in the specimens likely makes them effective nucleation sites, facilitating equiaxed grain formation. M. Dada et al. [192] investigated the influence of laser parameters on microstructural characteristics and hardness properties of DED-processed AlCoCrFeNiCu and AlTiCrFeCoNi HEAs. The results demonstrated a substantial increase in microhardness: the AlCoCrFeNiCu alloy exhibited a 300% rise and the AlTiCrFeCoNi alloy a 70% increase as the laser power escalated from 600 W to 800 W. Conversely, the microhardness decreased with higher scanning speeds. Microscopic analysis revealed a columnar dendritic structure for AlCoCrFeNiCu and equiaxed dendrites with oriented grain growth for AlTiCrFeCoNi, influenced by titanium content promoting strength through solid laves phase formation.

N. Malatji et al. [183,185] found that the AlCrFeNiCu HEA fabricated via LMD exhibited a dendritic microstructure comprising dual-phase (BCC + FCC) solid solutions. The results indicated that the wear resistance decreased with increasing laser power, with worn surfaces showing adhesive wear characteristics [183]. Heat treatment induced significant microstructural changes, and the alloy heat treated at 950 °C demonstrated improved wear resistance [185].

J. Joseph et al. [189] employed DLF to fabricate bulk samples of AlxCoCrFeNi HEAs, with varying Al concentrations of 0.3, 0.6, and 0.85 M fraction, featuring FCC, duplex FCC + BCC, and BCC crystal structures, respectively. The strength of the AlxCoCrFeNi HEAs was found to increase with an increase in the Al concentration, but this was at the expense of ductility. The high strength of the BCC alloy, over 2 GPa, has been attributed to its high volume fraction of spinodal second phase particles. A. Mohanty et al. [188] investigated the application of DED for fabricating HEAs, specifically Al_0.3_CoCrFeNi and Al_0.7_CoCrFeNi, known for their BCC and FCC plus BCC crystal structures, respectively. Al_0.3_CoCrFeNi displayed higher mass gain compared to Al_0.7_CoCrFeNi during cyclic oxidation at 1100 °C for 200 h. Oxidized surfaces of both alloys formed external Cr_2_O_3_ scales with underlying Al_2_O_3_ sub-scales, where the thickness and continuity of these oxide layers varied with the Al content. The findings suggest that a higher Al content contributes to improved oxidation resistance in these HEAs. B. Gwalani et al. [165] studied DED-fabricated laminates of Al_0.3_CoCrFeNi and Al_0.7_CoCrFeNi, finding that Al0.3CoCrFeNi exhibited a single-phase FCC structure, whereas Al_0.7_CoCrFeNi showed a dual-phase FCC + B2 structure. H. Peng et al. [182] investigated the microstructure and mechanical properties of Al_0.3_CoCrFeNi HEA fabricated using LMD. The as-built HEA exhibited a <110> fiber texture oriented along the build direction, accompanied by a high density of dislocation loops and neighboring dislocations. Thermal stresses induced dislocation glide and disorder-to-order transformations during LMD, leading to the formation of precipitated B2 particles during cooling or subsequent thermal exposure. Cracks tended to initiate and propagate along the boundaries between the FCC matrix and precipitated B2 phases, reducing ductility. Subsequent annealing promoted further precipitation of the B2 and σ phases, enhancing hardness and wear resistance, while also facilitating dislocation recovery and reducing the yield stress. M.S.K.K.Y. Nartu et al. [163] processed an Al_0.3_CoCrFeNi HEA using LENS, revealing the formation of nanometer-scale Al–Ni-rich solute clusters in the as-processed Al_0.3_CoCrFeNi HEA samples, attributed to re-heating effects during AM. Heat-treated samples showed even higher YS due to precipitation strengthening from nanometer-scale L1_2_ precipitates. Jithin Joseph et al. [186] investigated the tension/compression behavior of the DED-manufactured FCC Al_0.3_CoCrFeNi HEA and observed notable tension/compression asymmetry in both the work hardening rate and ductility of the alloy. The alloy exhibited exceptionally high work hardening under compression, attributed to extensive mechanical twinning exacerbated by a pronounced as-cast texture. In contrast, the material which deformed under tension showed minimal work hardening and reduced ductility, operating predominantly via slip mechanisms without significant mechanical twinning.

Q. Wang et al. [181] found that increasing the laser power significantly influenced the morphology of columnar crystals in LMD-processed CoCrFeNiMo_0.2_ HEAs, resulting in larger columnar grain sizes due to reduced temperature gradients. Tensile properties at 293 K were found to be adjustable by varying the laser power, corresponding to changes in microstructure. When the temperature was decreased from 293 K to 77 K, there was a significant enhancement in both the tensile strength and ductility of LMD-processed CoCrFeNiMo_0.2_ HEAs by approximately 70% and 28%, respectively, with the tensile strength reaching 928 MPa and the ductility reaching 60%. In the study conducted by K. Zhou et al. [191], uniform and fully compact microstructures were noticed in both the longitudinal and transverse sections of CoCrFeNiNbx HEA. There were no apparent pores or microcracks in any of the specimens. Moreover, an evolution from a columnar to an equiaxed grain structure was witnessed as the Nb content increased. Room-temperature tensile tests demonstrated that these alloys exhibited superior strength and ductility in comparison to their as-cast counterparts.

I. Kunce et al. [170] synthesized a ZrTiVCrFeNi HEA using LENS. Microstructural analysis revealed a dual-phase structure with a dominant C14 Laves phase matrix and a minor α-Ti solid solution phase. Hydrogen storage properties were evaluated via pressure–composition–temperature (PCT) isotherms up to 100 bar at 50 °C, demonstrating a maximum hydrogen capacity of 1.81 wt% post-synthesis and 1.56 wt% after additional annealing. A persistent C14 hydride phase was observed after PCT testing. Dobbelstein et al. [172] employed LMD to fabricate compositionally graded refractory HEAs through in-situ alloying of elemental powder blends, achieving a gradient ranging from Ti_25_Zr_50_Nb_50_Ta_25_ to Ti_25_Zr_0_Nb_50_Ta_25_ by progressively substituting Zr powder with Nb powder. Compositions ranging from Ti_25_Zr_0_Nb_50_Ta_25_ to Ti_25_Zr_25_Nb_25_Ta_25_ were identified as single-phase BCC solid solutions exhibiting coarse-grain microstructures. Increasing the Zr to Nb ratio yielded finer, harder multiphase microstructures. H. Dobbelstein et al. [178] employed LMD to fabricate a TiZrNbHfTa HEA from elemental powders, achieving a near-equiatomic composition and a BCC single-phase microstructure with uniform grain size and equiaxed morphology. The as-built specimen exhibited a high hardness of 509 HV0.2. I. Kunce et al. [161] employed LENS to synthesize TiZrNbMoV HEAs, revealing that varying laser powers (300 W and 1 kW) significantly influenced the microstructure and hydrogen storage properties. The alloys synthesized with different parameters exhibited distinct morphologies and phases, including a dendritic matrix with unmelted Mo particles and a multi-phase microstructure enriched in Mo and Zr, which affected their hydrogen absorption and desorption behaviors. H. Dobbelstein et al. [158] explored DMD of an equimolar mixture of elemental powders using a pulsed Nd: YAG laser, aiming to investigate the deposition strategies and microstructure of MoNbTaW. Their study analyzed the formed single-wall structures, highlighting the capability of DMD to generate composition gradients through dynamic powder mixing, which circumvents the limitations of pre-alloyed powders.

There are some studies on the preparation of HEA composites using DED. Q. Shen et al. [196] utilized PPAAM to fabricate CoCrFeNi(SiC)x (x = 0, 0.1, 0.3, and 0.5) HEAs, revealing that an increase in SiC content led to a transformation in microstructure from a single FCC phase to an FCC + Cr_7_C_3_ dual-phase, accompanied by significant enhancements in hardness (from ~139 HV to ~310 HV) and YS (from ~142 MPa to ~713 MPa). These enhancements were attributed to solid-solution and second-phase strengthening mechanisms. J. Li et al. [177] developed an LMD technique incorporating WC addition to produce high-strength CrMnFeCoNi-based HEA composites, enabling the formation of a microstructure characterized by compact refined equiaxed grains. Specifically, the sample incorporating 5 wt% WC exhibited promising mechanical properties, including a tensile strength of 800 MPa and an elongation of 37%. The improved mechanical performance is attributed to the formation of Cr_23_C_6_ reinforcement precipitates, which facilitate heterogeneous nucleation of grains and impede the propagation of slip bands within the alloy matrix. Amar et al. [184] demonstrated the fabrication of high-strength CrMnFeCoNi-based HEAs using LMD, with the tensile properties tailored through the controlled addition of TiC. Specifically, the incorporation of 5 wt% TiC into the CrMnFeCoNi HEA led to a tensile strength of 723 MPa and a tensile strain of 32%. This enhancement in mechanical performance was attributed to the introduction of micron-sized TiC reinforcement phases, which effectively inhibited the propagation of slip bands within the alloy microstructure. S. Guan et al. [171] additively manufactured a laminated HEA combining CrMnFeCoNi and AlCoCrFeNiTi_0.5_, featuring alternating layers of these constituent materials. The composite HEA exhibited exceptional strength–plasticity synergy under compression, achieving a YS of up to 990 MPa with no complete fracture observed until 80% strain, surpassing the performance of monolithic bulk HEAs. This synergy is attributed to heterogeneous microstructures consisting of ultra-hard BCC equiaxed grains in the AlCoCrFeNiTi0.5 lamellae and soft FCC columnar grains in the CrMnFeCoNi lamellae, arranged periodically. Y. Cai et al. [179] fabricated a laminated FeCoCrNi + FeCoCrNiAl HEA using LMD, highlighting distinct microstructural and mechanical properties. The FeCoCrNi component exhibited a BCC phase with exceptional ductility and toughness, while the FeCoCrNiAl alloy showed higher strength, albeit with reduced toughness due to its BCC phase. The combined laminated HEA demonstrated superior ductility, strength, and enhanced crack resistance, offering potential advancements for diverse industrial applications.

Additionally, DED has been developed as a high-throughput synthesis technique for the screening and design of HEAs, including CoCrFeMnNi [176], AlCrFeMoV [169], MoNbTaW [197,198], Nb*_x_*CoCrFeMnNi [154], Ta_x_CoCrFeMnNi [154], and (TiAl_6_V_4_)*_x_*CoCrFeMnNi [154].

### 2.5. Binder Jetting (BJT)

**Figure 4:**
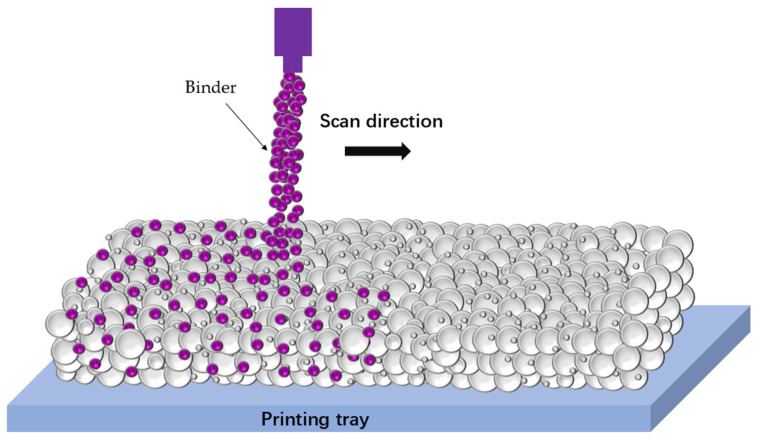
Schematic of typical BJT process.

BJT is an AM process where a liquid bonding agent is selectively deposited to bind powder materials together [41]. It makes use of a powder bed system and utilizes a liquid binder rather than a heat source, as shown in Figure 4. This method involves applying a binder material onto a powder bed to create components layer by layer, resulting in a “green body” that requires sintering to achieve sufficient density. Consequently, post-processing steps, such as sintering or infiltration, are often necessary to attain the final material properties. Key characteristics of this technique include its ability to create complex geometries, accommodate a wide range of materials (such as metals, ceramics, and polymers), and generally build faster speeds compared to some other AM methods.

Only three papers were found regarding HEAs manufactured using binder-based AM techniques. D. Karlsson et al. [199] fabricated high-density components of an AlCoCrFeNi alloy through BJT and subsequent sintering. The as-sintered material exhibited a predominant B2/BCC structure with additional FCC phases and sigma phase precipitates. Annealing experiments at temperatures between 1000 °C and 1100 °C suppressed the sigma phase and led to a microstructure dominated by B2/BCC and FCC phases. Higher annealing temperatures resulted in further enhanced mechanical properties, demonstrating increased yield and fracture strengths. L. Chen et al. [201] explored the influence of sintering temperature and hot isostatic pressing (HIP) on the microstructure, mechanical properties, and electrochemical behavior of equiatomic FeNiCoCr HEA samples produced via BJT. The sintered samples exhibited a single FCC phase, with relative densities increasing with higher sintering temperatures. HIP substantially enhanced both the densities and mechanical properties of samples. The formation of Co(Fe, Cr)_2_O_4_ in the passive film contributed to the improved corrosion resistance of FeCoNiCr. Z. Xu et al. [200] investigated the BJT process for manufacturing porous CoCrFeMnNi HEA. Porosities ranging from 35% to 40% were achieved through BJT and subsequent sintering processes. X-ray computed tomography measurement indicated that the morphology and size of the pores were uniformly distributed. Fracture surface investigations revealed transgranular quasi-cleavage fractures to be more dominant. The corrosion resistance of the porous CoCrFeMnNi was found to be comparable to the 316 L equivalent upon optimum sintering parameters.

### 2.6. Direct Ink Writing (DIW)

**Figure 5:**
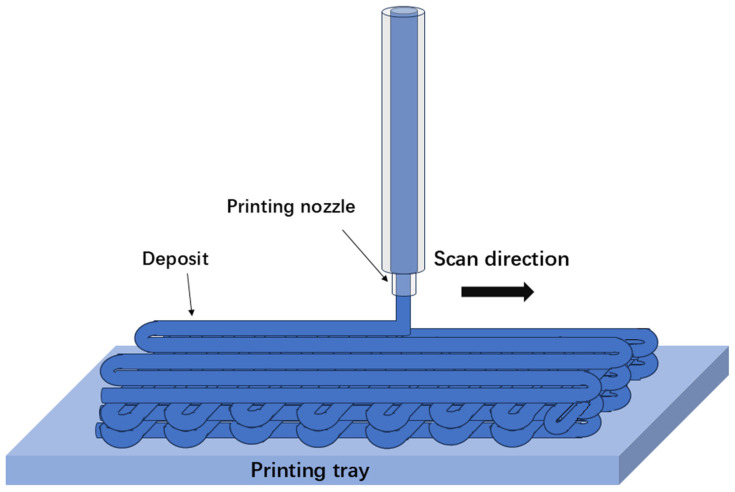
Schematic of typical ME process.

Direct ink writing (DIW) is another type of AM process. It begins with the preparation of a specially formulated ink or paste material. This material typically has a suitable viscosity that allows it to flow smoothly through a nozzle. The ink is then loaded into a dispensing system, which extrudes the ink in a controlled manner according to a pre-programmed pattern during the manufacturing process. The material is deposited layer by layer, either onto a substrate or onto previously deposited layers, as shown in Figure 5. The deposition can be executed in a planar fashion or a more complex 3D path, enabling the creation of intricate geometries. After deposition, the printed part may undergo post-processing steps such as curing or sintering to enhance its mechanical properties and finalize its structure.

C. Kenel et al. [202] developed a method for AM of HEAs aimed at integrating their unique mechanical properties with the geometric complexity required in modern designs. This technique involves the 3D extrusion of inks containing a blend of oxide nanopowders (Co_3_O_4_ + Cr_2_O_3_ + Fe_2_O_3_ + NiO), followed by co-reduction to metals, inter-diffusion, and sintering under H_2_ atmosphere to achieve a near-full-density CoCrFeNi alloy. A complex phase evolution where oxide phases undergo reduction leads to inter-diffusion of the resulting metals and the formation of FCC equiatomic CoCrFeNi alloy. This phase evolution is accompanied by structural changes from loosely packed oxide particles in the initial green body to fully annealed metallic CoCrFeNi with 99.6 ± 0.1% relative density. The fabricated CoCrFeNi micro-lattices exhibit strut diameters as small as 100 μm and demonstrate excellent mechanical properties at both ambient and cryogenic temperatures. S. Peng et al. [203] produced 3D-architected CoCrFeNiMn HEAs through DIW and thermal sintering. The resulting 3D-architected CoCrFeNiMn structures displayed remarkable energy absorption capabilities, surpassing conventional architected materials. This enhanced energy absorption was attributed to the bend-dominated deformation mode of the 3D architecture and the fully annealed homogeneous microstructure characterized by equiaxed grains, contributing to significant strain hardening during deformation.

### 2.7. Additive Friction Stir Deposition (AFSD)

**Figure 6:**
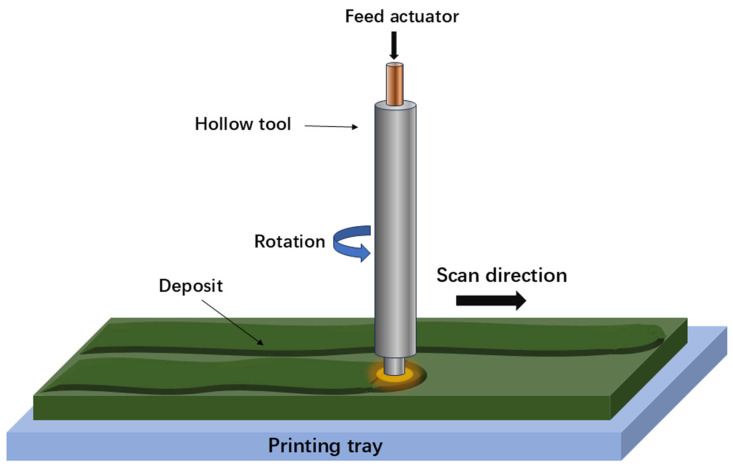
Schematic of typical AFSD process.

Additive friction stir deposition (AFSD) is an advanced manufacturing technique that combines the principles of friction stir welding with additive manufacturing, as shown in Figure 6. During the AFSD process, a rotating tool generates frictional heat to deposit material layer by layer, which makes it possible to create complex geometries and structures, especially those made of metals. This approach offers significant advantages, including enhanced material properties due to localized heating and mechanical stirring, which contribute to the formation of strong, high-quality deposits with minimal porosity. AFSD is particularly useful for applications demanding high-strength materials and intricate designs. However, the process can introduce residual stresses into the material, which may lead to cracking or deformation over time. Additionally, the surface finish of the deposited material may not be as smooth as with some other AM processes, requiring additional post-processing steps.

G.M. Karthik et al. [204] employed AFSD to fabricate a metal–metal composite comprising an AA5083 alloy reinforced with nanocrystalline CoCrFeNi HEA particles. The interfaces between layers and reinforcement/matrix interfaces exhibited no formation of brittle intermetallic compounds, attributed to the inert nature, high strength, and hardness of the HEA particles. The composite demonstrated significantly improved tensile and compressive strengths compared to the standard wrought-processed alloy AA5083-H112 while offering improved ductility. Similarly, P. Agrawal et al. [205] explored AFSD as a solid-state AM technique capable of producing defect-free 3D components with homogeneous, equiaxed microstructures. The AFSD-processed Fe_40_Mn_20_Co_20_Cr_15_Si_5_ HEA exhibits transformation and twinning-induced plasticity during deformation. Intense shear deformation at elevated temperatures and strain rates during AFSD-induced mechanisms such as recovery, recrystallization, and grain growth resulted in refined grains with enhanced strength and work hardenability.

## 3. HEAs Powders and Wires for AM

In the field of AM, metal powders and wires are essential raw materials that enable the fabrication of complex and high-performance components [219]. Each material type is tailored to specific AM technologies, significantly influencing the final product’s quality and functionality [220]. Metal powders are primarily used in PBF processes such as SLM and SEBM and are typically manufactured through atomization techniques, with gas atomization being the most common. This process entails the rapid dispersion of molten metal into fine droplets by means of a high-velocity stream of inert gas, and then these droplets solidify into spherical powder particles [221]. Alternative methods, such as water atomization and centrifugal atomization, also produce metal powders with varying characteristics suited to different applications. Fine powders with narrow size distributions are often preferred for their ability to produce components with excellent mechanical properties and minimal porosity. There are two main approaches for preparing composite powder: one involves melting a composite ingot and atomizing it to produce the powder, while the other involves mixing pre-powdered matrix alloys with metallic elemental powders or nano- or micron-sized reinforced particles [112,177]. The latter approach is often more practical due to the high cost of producing tailored atomized pre-alloyed powder compared to cast and wrought alloys [222,223]. Metal wires, on the other hand, are utilized in wire-feed AM processes such as WAAM [224]. Metal wires are typically produced through an extrusion or drawing process, where bulk metal is drawn through a series of dies to achieve the desired diameter and mechanical properties. Both metal powders and wires require precise handling and processing to maintain their quality and effectiveness in AM [220]. For powders, attributes like particle size and distribution affect flowability and layer density, while for wires, consistency in diameter and composition impacts deposition reliability and part quality [219].

Currently, gas atomization is the leading technology for producing metal powders for AM, including HEA powders. This method can produce HEA powders with particle sizes in the range of tens of micrometers, such as CoCrFeNi powder (~20–63 μm) [74], CoCrFeMnNi powder (34.0 μm) [225], and CoCrFeNiC_0.05_ powder (~45 μm) [117]. In the literature reviewed on HEAs in AM, the majority of studies explicitly state that the used powders were produced using gas atomization [70,71,73,74,75,76,77,80,81,82,85,88,89,91,92,93,109,110,112,114,115,116,117,118,120,121,125,126,127,128,129,130,131,133,134,137,141,142,144,146,147,148,149,151,153,155,160,161,162,164,165,166,168,170,174,175,178,179,180,181,182,187,189,194,195,199,200,201,217,218,225,226,227,228]. Among the remaining papers, except for some that used commercially available powders without specifying the production method, only a few employed methods other than gas atomization. HEAs such as Ni_2.1_CoCrFeNb_0.2_, CrMnFeCoNi, and CoCrFeNiNb_0.1_ were prepared using the plasma rotating electrode process (PREP) [135,167,191]. Additionally, T. Na et al. [229] successfully produced spherical TaNbHfZrTi HEA powders through a hydrogenation–dehydrogenation reaction and thermal plasma treatment, achieving a transformation from irregularly shaped powders with multiple phases to uniformly spherical powders with a single solid-solution BCC phase. J. Park et al. [230] prepared spherical WTaMoNbV refractory HEA powders through milling and spheroidization via inductively coupled thermal plasma, resulting in a transformation from irregularly shaped powders to spherical particles with an average size of d50 = 45.1 µm.

## 4. Post-Processing of AM-Processed HEAs

AM processes produce net-shape products, limiting the application of work hardening methods to improve mechanical properties. Consequently, AM-manufactured components are typically used either in the as-deposited state or after heat treatment. Additionally, techniques such as HIP and laser shock peening (LSP) have been employed to improve the performance of AM-manufactured components.

Heat treatment notably influences the mechanical properties of AM-processed HEAs through mechanisms such as residual stress relief, phase precipitation, and grain growth. T. Fujieda et al. explored the effects of solution treatment, during which specimens were heated at 1393 K, on AM-processed CoCrFeNiTi-based HEAs. They achieved enhanced tensile strength and corrosion resistance by combining SLM or SEBM with solution treatment [130,151]. These improvements were attributed to the homogeneous precipitation of fine Ni–Ti-rich particles during solution treatment [130,151]. D. Lin et al. [71] investigated the effects of annealing on SLM-processed FeCoCrNi HEAs. They found that annealing at temperatures of 773 K, 873 K, 973 K, 1073 K, 1173 K, 1273 K, 1373 K, 1473 K, and 1573 K for 2 h and then cooling in a furnace led to significant microstructural changes, such as recrystallization from columnar to equiaxial grains, along with numerous annealing twins. This process reduced residual stress, yield strength, and hardness while improving plasticity and impact toughness, thereby enhancing industrial applicability. D. Vogiatzief et al. [116] explored the heat treatment of AlCrFe_2_Ni_2_ HEA. They carried out post-build annealing heat treatments at 750, 800, and 850 °C for 3 h as well as at 900 and 950 °C for 6 h in order to examine the effects of temperature and treatment time on the microstructure. The specimens were heat-treated in a furnace under argon at a heating rate of 1.1 °C/s and then cooled down to room temperature inside the furnace at a cooling rate of approximately 0.5 °C/s. It was discovered that heat treatment of AlCrFe_2_Ni_2_ HEA transformed an initially metastable state into an ultrafine duplex microstructure by using spinodally decomposed BCC phases to nucleate FCC micro-platelets. Zhang et al. [127] performed heat treatment on the SLM-fabricated AlCoCuFeNi HEA samples at 900 °C and 1000 °C for 10 h, respectively. Prior to that, they sealed the samples beforehand in vacuum-encapsulated quartz tubes to prevent oxidation during the heat treatment. They observed that the heat treatment led to the precipitation of a Cu-rich BCC phase, resulting in a dual-phase microstructure. This thermal processing reduced microhardness and compressive yield strength while significantly enhancing ductility compared to the as-fabricated state, with the precipitation of the FCC phase contributing to improved toughness and strain hardening. V.V. Popov et al. [150] carried out heat treatment, aiming at microstructural homogenization, for 24 h within vacuumed quartz ampules under two different conditions, specifically at 1000 °C and 1300 °C. They found that, although it was possible to obtain a lower porosity level by means of heat treatment at 1300 °C for 24 h, this approach led to unfavorable changes in the alloy’s composition. Furthermore, they emphasized that the SEBM production of Al_0.5_CrMoNbTa_0.5_ HEAs from elemental powder blends is possible, and optimizing process parameters rather than relying on post-process heat treatments is essential to mitigate porosity in the samples. W.C. Lin et al. [131] explored the microstructure and tensile property of Al_0.2_Co_1.5_CrFeNi_1.5_Ti_0.3_ HEA fabricated by SLM, as well as those of the alloy processed by SLM followed by direct aging at 750 °C for 50 h and then air-cooled to room temperature. The combination of SLM and post-heat treatment remarkably enhanced the tensile properties at both room temperature and 500 °C. The sample processed by SLM along with direct ageing acquired additional strengthening effects from finer grain size, nano oxides, internal stress, and the L21 phase with a subgrain structure. The yield strength (YS) and ultimate tensile strength (UTS) of Al_0.2_Co_1.5_CrFeNi_1.5_Ti_0.3_ HEA processed by SLM plus direct ageing were 1235 MPa and 1550 MPa, respectively, and they were the highest to date for HEAs subjected to laser AM [131].

J. Joseph et al. [190] investigated the impact of HIP on the density, microstructure, and mechanical properties of an AM-prepared AlxCoCrFeNi system with aluminum molar fractions (x) of 0.3, 0.6, and 0.85, featuring FCC, duplex FCC + BCC, and BCC crystal structures, respectively. HIP was found to reduce the presence of large pores (>5 µm) and marginally increase alloy density, while also causing microstructural coarsening and chemical homogenization. HIP improved the mechanical properties of FCC HEA (x = 0.3). Although it enhanced the compressive properties of the dual-phase HEA (x = 0.6), it weakened the tensile properties due to the coarsening of hard BCC grain boundary precipitates. In the high-aluminum HEA (x = 0.85), mechanical properties were negatively impacted by σ-phase formation at the phase and grain boundaries, leading to brittle fractures in both tension and compression. In the study of R. Li et al. [88], HIP was performed on the SLM-processed equiatomic CoCrFeMnNi HEA to eliminate the metallurgical defects. The HIP-treated HEA presented a higher densification and relatively large grains. It closed most micropores and microcracks, increasing the relative density from 98.2% before HIP to 99.1% after HIP. The tensile strength increased from 601 MPa to 649 MPa while the elongation decreased from 35.0% to 18%. The HIP-treated sample had a lower residual stress than the SLM-printed sample, owing to the non-equilibrium processing of SLM and the distressing effect of HIP treatment. After HIP, the preferential orientation became less significant as compared with the SLM samples.

In a study by Z. Tong et al. [156], LSP was employed to change the stress state and microstructure of the surface layer of AM-fabricated CrMnFeCoNi HEA and improve its mechanical properties. The results indicated that LSP transformed the surface-stress state from tensile to compressive stress, and the pores in the surface layer were closed after undergoing severe plastic deformation. Additionally, LSP resulted in the development of a gradient microstructure along the depth direction. The formation of a sandwich structure with a hardened surface layer and soft core improved the strength and ductility of the specimens treated by LSP. The 1 LSP specimen reached 427.4 MPa, 570.7 MPa, and 40.1%, which were approximately 33.3%, 7.3%, and 15.13% greater than those of the as-built specimen (YS-320.7 MPa, UTS-531.7 MPa, and elongation—31.9%), respectively. When the number of impacts increased to three and five, the YS, UTS, and elongation values further improved. Notably, the five LSP specimens exhibited the highest YS (489.8 MPa), UTS (639.9 MPa), and elongation (61%) values.

## 5. Mechanical Properties of AM-Processed HEAs

Unlike conventional manufacturing techniques, AM technologies achieve unique ultra-fine and non-equilibrium microstructures through highly localized melting, strong temperature gradients, and extremely high cooling rates, resulting in superior mechanical properties such as enhanced strength and ductility. Compared to conventionally cast HEAs, the AM-fabricated HEAs exhibited significantly elevated strength [146,147,151,153,155,162,191]. Nevertheless, there are also certain challenges associated with these technologies. For instance, improving the fatigue strength of the AM-fabricated AlSi_10_Mg alloy constitutes one of the challenges [231]. In cyclic tests, substantial anisotropy is present and the cyclic properties deteriorate. Despite technological progress, the current AM technology is unable to attain the same degree of cyclic property isotropy as that of convectively cast material because of physical limitations.

The strength and the elongation of HEA with FCC crystal structure far exceed those of HEAs with BCC crystal structure, such as AlCrCuFeNi or AlCoCuFeNi fabricated by SLM. This disparity might occur because BCC-structured HEAs are more prone to cracking than those with an FCC structure. The FCC structure has 48 slip systems, in contrast to the BCC structure, which has only 12 slip systems. This explains why the elongation of FCC HEAs processed by SLM is higher than that of BCC HEAs. It is worth noting that the FeMnCoCrNi HEA, which has an FCC structure, exhibits high elongation [92].

**Figure 7:**
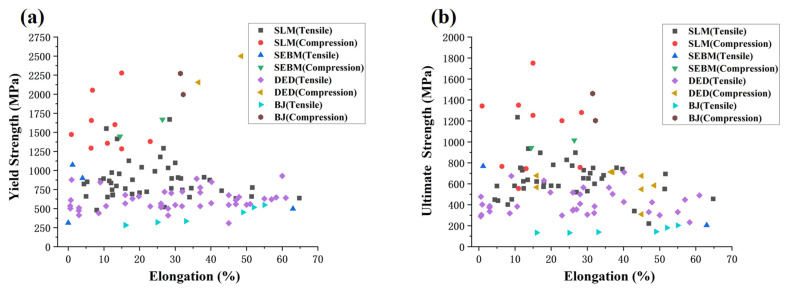
Strength vs. elongation using various AM methods. (**a**) YS vs. elongation, (**b**) UTS vs. elongation.

Figure 7 shows the strength and ductility obtained from both compression and tension experiments on AM-processed HEAs, with specific alloy compositions and properties listed in Table 1. Comparing the performances of HEAs produced by SLM and DED, it is observed that SLM-processed HEAs generally exhibit higher strength than those produced by DED. In contrast, HEAs produced by BJT have the lowest tensile strength, although their compressive strength is relatively high.

In addition to tensile and compressive testing, research on the fatigue properties, crack formation, and creep deformation behavior of HEAs remains relatively limited [72,80]. Y.O. Kuzminova et al. [72] explored the fatigue properties of CrFeCoNi alloys fabricated via AM and found that the machined samples exhibited significantly higher fatigue resistance in both as-built and as-annealed conditions. As-built samples, without annealing, can endure 10^7^ cycles at a maximum fatigue stress of 414 MPa (stress amplitude (σ_a_) of 186.3 MPa), whereas unmachined samples reach a maximum fatigue stress of only 138 MPa (σ_a_ = 62.1 MPa). Heat treatment generally improves fatigue life under high-cycle fatigue conditions for both machined and unmachined alloys. Z. Xu et al. [80] examined the nanoindentation creep deformation behavior of SLM-fabricated CoCrFeMnNi HEAs under varying peak holding loads, revealing that creep deformation was predominantly governed by dislocation motion. C. Zhang et al. [91] studied crack formation and mitigation strategies, finding that cracks mainly initiate along right-angle-shaped HAGBs without elemental segregation. The upper crack portion shows hot cracking characteristics, while the lower likely forms during solid-state propagation due to thermal shrinkage.

The scanning strategy significantly affects crack morphology. However, P. Niu et al. [92] found that SLM-printed CoCrFeMnNi HEA experienced hot cracking regardless of printing parameters. The SLM-fabricated CrMnFeCoNi HEA showed excellent resistance to hydrogen embrittlement, attributed to its high hydrogen solubility and easy formation of deformation twins during loading [86]. YS was also confirmed to increase further after H-charging due to interstitial strengthening [87]. Y.K. Kim et al. [83] investigated the high-temperature creep behavior of equiatomic CoCrFeMnNi HEA fabricated via SLM. M. Jin et al. [84] explored the cyclic plasticity and fatigue damage behavior of an SLM-processed CrMnFeCoNi alloy, finding that plastic deformation under monotonic loading involved both dislocation slip and deformation twinning, with dislocation slip identified as the predominant mechanism during cyclic loading. Varying scan strategies influenced grain morphology and size, impacting fatigue crack propagation behavior. Notably, the meander scan strategy with 0° rotation exhibited enhanced resistance to crack propagation due to its promotion of columnar grains and minimized grain boundary spacing along the crack path.

## 6. Corrosion Performances of AM-Processed HEAs

Due to the high mixing entropy effect, HEAs tend to form disordered solid solutions with FCC, BCC, or HCP structures, rather than intermetallic compounds typical of conventional alloys. In conventional alloys, the matrix surrounding the intermetallic compounds acts as a micro-anode, corroding preferentially, while the intermetallic compounds function as micro-cathodes. Furthermore, HEAs containing passivating elements such as Cr, Ni, and Mo demonstrate superior corrosion resistance compared to conventional alloys by forming protective oxides. Additionally, AM improves the compositional uniformity of HEA, mitigating compositional segregation that can lead to galvanic and pitting corrosion. This uniform composition facilitates the formation of a consistent passivation film, thereby enhancing corrosion resistance. For example, a comparative analysis of CoCrFeMnNi HEAs produced by SLM and as-cast methods in a 3.5 wt% NaCl solution revealed that the SLM-produced HEA exhibited a more uniform composition and finer grains, resulting in a passivation film with enhanced stability and protective properties [232]. Consequently, the corrosion current density of the SLM-produced HEA decreased by 58% compared to the as-cast HEA, indicating significantly better corrosion resistance. J. Ren et al. [227] investigated the corrosion resistance of SLM-manufactured CoCrFeMnNi HEAs in 3.5 wt% NaCl solution using potentiodynamic polarization and electrochemical impedance spectroscopy tests. Compared with their conventional as-cast counterparts, the AM CoCrFeMnNi HEAs showed higher pitting resistance (ΔE) and greater polarization resistance (Rp). The superior corrosion resistance of AM-processed CoCrFeMnNi HEAs may be attributed to the homogeneous elemental distribution and lower density of micro-pores. However, H. Peng et al. [226] found that the SLM-printed HEA exhibited inferior corrosion resistance in a 3.5 wt% NaCl solution compared to the cast counterpart, attributed to the higher density of dislocations, pores, and cracks in the SLM samples, which significantly compromised their corrosion resistance. Q. Wang et al. [181] utilized LMD to fabricate CoCrFeNiMo_0.2_ HEAs and investigated their corrosion behavior under varying laser powers. The study revealed that the CoCrFeNiMo_0.2_ alloy exhibited superior corrosion resistance compared to 304 stainless steel substrate and CoCrFeNi, as well as an even lower corrosion current density than 316L stainless steel, in both 3.5 wt% NaCl solution and 1 mol/L H_2_SO_4_ solution.

Mass loss tests, polarization tests, and electrochemical impedance spectroscopy were employed to assess the corrosion resistance properties of AM-processed HEAs, whereas first principle calculations were utilized to gain insights into the corrosion resistance by predicting formation energies, secondary phase precipitation, and the development of intermetallic phases [132,143,226,227]. Additionally, to address the demands of challenging industrial environments for HEA applications, unconventional tests were also conducted. For example, the elevated temperature corrosion resistance of SLM-processed AlCoFeNiTiV_0.9_Sm_0.1_ and AlCoFeNiV_0.9_Sm_0.1_ HEAs was evaluated in a harsh corrosive syngas atmosphere at elevated temperatures to simulate challenging industrial conditions [132].

The corrosion properties of AM-processed HEAs are influenced by alloy microstructure, defects, composition and processing conditions. H. Peng et al. investigated how corrosion resistance varied between different orientations of the SLM-printed samples relative to the building direction. Electrochemical anode dissolution rates were calculated for the SLM-printed CoCrFeMnNi HEAs in orientations parallel and perpendicular to the building direction, revealing that the presence of pores and cracks exerted a more pronounced influence on corrosion resistance than grain boundaries and grain orientation [226]. T. Fujieda et al. [130] found the SLM-processed CoCrFeNiTi-based HEA to have a fine and uniform microstructure without visible segregation. This microstructural refinement contributed to a higher pitting potential (0.88 ± 0.03 V vs. Ag/AgCl in 3.5% NaCl solution at 353 K) compared to EBM counterparts (0.50 ± 0.04 V vs. Ag/AgCl, respectively). The as-built and solution-treated SLM specimens exhibited exceptional resistance to pitting corrosion compared to traditional highly corrosion-resistant alloys. The study also examined the effect of solution treatment, revealing that water quenching significantly improved the pitting–corrosion resistance of the SLM specimens. M. A. Melia et al. [168] investigated the corrosion properties of AM-processed CoCrFeMnNi HEAs. The annealing treatment resulted in a recrystallized microstructure with improved elongation and corrosion resistance. Corrosion behavior revealed preferential attacks on inter-cellular Ni/Mn regions, suggesting a complex pit morphology. T. Fujieda et al. [151] successfully fabricated a Co_1.5_CrFeNi_1.5_Ti_0.5_Mo_0.1_ HEA with enhanced corrosion resistance through SEBM combined with subsequent solution treatment.

The AM process may introduce residual stresses that could cause local microstructural changes and defects, such as dislocations, potentially affecting the corrosion properties of HEAs. Stress corrosion cracking (SCC) behavior in AM-processed HEAs is another important aspect, as AM components are known to possess residual stresses due to the unique thermal cycle involved in the process. However, no studies on SCC behavior in AM-HEAs have been reported in the literature to date [233].

## 7. Summary and Prospects

This paper offers a thorough review regarding the application of AM in the fabrication of HEAs. AM has emerged as a promising technique for manufacturing HEAs, facilitating the production of complex geometries and customized properties. Recent progress in AM technologies has witnessed significant achievements in the production of HEAs. A variety of AM methods, including SLM, SLS, SEBM, DED, BJT, DIW, and AFSD, have been investigated for processing HEAs. These technologies have made it possible to fabricate HEAs with distinctive microstructures, resulting in improved mechanical properties that exceed those of conventional alloys. The strength and ductility of HEAs produced via AM techniques have been especially remarkable, as some alloys have shown a combination of high strength and good ductility, which is difficult to attain using traditional manufacturing processes.

SLM is the most extensively studied AM technique for HEAs, and it can produce fine and uniform microstructures with adjustable properties by controlling processing parameters. Other AM techniques also have their unique features and applications in fabricating HEAs. The powders and wires used in AM play a crucial role in the final quality of the fabricated components. Gas atomization is commonly used to produce metal powders for AM, including those used for HEAs. Post-processing techniques like heat treatment, HIP, and LSP can improve the performance of AM-processed HEAs. AM-processed HEAs exhibit superior mechanical properties; however, research into their fatigue and creep behavior remains relatively scarce. The corrosion properties of AM-processed HEAs are influenced by multiple factors, and AM has the potential to enhance the corrosion resistance by improving the composition uniformity.

Although AM technologies have achieved remarkable progress in manufacturing HEAs with better mechanical properties than those of conventional alloys, there remain unresolved challenges that must be tackled in order to fully unleash the potential of these materials. One of the principal issues lies in the variability of mechanical properties, which can be affected by multiple factors like printing parameters, post-processing treatments, and the inherent characteristics of the alloy itself. Another outstanding challenge pertains to the fatigue strength of AM-processed materials. The significant anisotropy present in AM-processed materials demands attention to guarantee the reliability and durability of these materials in practical applications. Moreover, further studies are essential to comprehensively understand the fatigue and creep behavior of AM-processed HEAs so as to broaden their potential applications in high-stress environments. Additionally, exploring the performance of HEAs in extreme environments is crucial in order to fulfill specific engineering requirements. This involves researching their behavior under high-temperature, high-pressure, and corrosive conditions, among others. Furthermore, the development of efficient and cost-effective post-processing methods will also play a vital role in improving the performance and reliability of AM-processed HEAs.

Future research in this field may concentrate on further optimizing the AM processes to achieve better control over the microstructure and properties of HEAs. This could involve refining processing parameters, exploring new alloy compositions, and improving the quality and consistency of the raw materials. Additionally, the integration of artificial intelligence and materials computation in the alloy design and process development of HEAs could accelerate the research and development process. By leveraging these technologies, it is possible to predict and optimize the properties of HEAs more efficiently, leading to the discovery of novel alloys and processing methods. Interdisciplinary cooperation among researchers, engineers, and industry experts is of great significance for speeding up the commercialization and practical application of AM-processed HEAs in a wide variety of fields, including aerospace, automotive, and biomedical industries.

Overall, the AM of HEAs holds substantial potential for the development of advanced materials equipped with distinctive and unique properties and functionalities. Continued research and innovation in this area will undoubtedly drive the progress of materials science and engineering.

## Figures and Tables

**Table 1:** Tensile and hardness results of the AM-processed samples.

Composition	AM	YS (MPa)	UTS (MPa)	Elongation (%)	Source
FeCoCrNi	SLM	402	480	8	[70]
FeCoCrNi	SLM	600	745	32	
FeCoCrNi	SLM	581.9	707.9	≈20	[71]
FeCoCrNi	SLM + annealing	221.0	633.2	≈47	
CoCrFeNi	SLM	556.7 ± 23.6	676.7 ± 20.5	12.4 ± 2.1	[74]
CoCrFeNi	SLM	572 ± 7.5	691.0 ± 15.9	17.9 ± 0.9	
CrFeCoNi	SLM	551.0 ± 9.6	658.3 ± 9.0	51.4 ± 10.9	[72]
CrFeCoNi	SLM + annealed at 800 °C	456.3 ± 9.9	637.5 ± 14.4	64.8 ± 3.7	
CoCrFeMnNi	SLM		681 ± 14	12.5 ± 0.5	[81]
CoCrFeMnNi	SLM	624 ± 4	747 ± 2	12.3 ± 0.2	[82]
CrMnFeCoNi	SLM	729.6	836.2	12	[86]
CrMnFeCoNi	SLM + gaseous H-charging	752.6	863.9	11.5	
FeCoCrNiC_0.05_	SLM	638	797	13.5	[118]
CoCrFeMnNiC_0.5_	SLM	653.0	766.3	28.9	[119]
CoCrFeMnNiC_1.0_	SLM	751.6	895.0	31.6	
CoCrFeMnNiC_1.5_	SLM	753.7	911.1	38.1	
CoCrFeMnNiC1 (X direction)	SLM	741	874	39.7	[120]
CoCrFeMnNiC1 (Y direction)	SLM	694	776	51.6	
CoCrFeMnNiC1 (Z direction)	SLM	681	768	34.5	
CoCrFeMnNiC1 (laser scanning speed: 200 mm s^−1^)	SLM	829	989	24.3	[121]
CoCrFeMnNiC1 (laser scanning speed: 600 mm s^−1^)	SLM	741	874	39.7	
FeCoCrNiSi_1.5_	SLM	701 ± 14	907 ± 25	30.8 ± 2	[124]
FeCoNiCr	SLM	520		27	[75]
FeCoNiCrN_1.8_	SLM	650		34	
FeCoCrNiMn-(N,Si) (scan rotations: 45°)	SLM	579	660	5	[126]
FeCoCrNiMn-(N,Si) (scan rotations: 67°)	SLM	622	763	16	
Fe_40_Mn_20_Co_20_Cr_15_Si_5_	SLM	530 ± 40	1100	30	[125]
Al_0.3_CoCrFeNi	SLM	730	896	29	[109]
Al_0.5_FeCoCrNi	SLM	579	721	22	[110]
Al_0.5_CoCrFeNi	SLM	609	878	18	[111]
AlCoCuFeNi	SLM	1342 (compressive)	1471 (compressive)	0.9 (compressive)	[127]
AlCoCuFeNi	SLM + annealed at 900 °C	766 (compressive)	1292 (compressive)	6.4 (compressive)	
AlCoCuFeNi	SLM + annealed at 1000 °C	744 (compressive)	1600 (compressive)	13.1 (compressive)	
AlCrCuFeNi	SLM (0°)		1655.2 ± 72.7 (compressive)	6.5 ± 0.7 (compressive)	[128]
AlCrCuFeNi	SLM (90°)		2052.8 ± 123.6 (compressive)	6.8 ± 1.3 (compressive)	
AlCrCuFeNi_3_	SLM		957	14.3	[217,218]
CoCrFeNiTi	SLM	773.0 ± 4.2	1178.0	25.8 ± 0.6	[130]
CoCrFeNiTi	SLM + water-quenched during solution treatment	897.5 ± 65.8	1291.0 ± 29.7	26.7 ± 2.3	
CoCrFeNiTi	SLM + air-cooled during solution treatment	935.5 ± 9.2	1414.5 ± 0.7	13.7 ± 0.4	
Al_0.2_Co_1.5_CrFeNi_1.5_Ti_0.3_	SLM	781	1042	20.58	[131]
Al_0.2_Co_1.5_CrFeNi_1.5_Ti_0.3_	SLM + aged at 750 °C for 50 h and then air cooled	1235	1550	10.69	
Ni_2.1_CoCrFeNb_0.2_	SLM	340	735	43	[135]
Ni_2.1_CoCrFeNb_0.2_	SLM + aged at 650 °C for 96 h	896	1127	17	
CoCr_2.5_FeNi_2_TiW_0.5_ (N_2_, X direction)	SLM	449	823	4.4	[136]
CoCr_2.5_FeNi_2_TiW_0.5_ (Ar, Y direction)	SLM	440	851	5.3	
CoCr_2.5_FeNi_2_TiW_0.5_ (N_2_, X direction)	SLM	452	869	9.1	
CoCr_2.5_FeNi_2_TiW_0.5_ (Ar, Y direction)	SLM	581	893	9.9	
Ni_6_Cr_4_WFe_9_Ti	SLM	742	972	12.2	[137]
NbMoTa	SLM	1252.56 (compressive)	1282.94 (compressive)	15 (compressive)	[140]
NbMoTaTi	SLM	1201.48 (compressive)	1380.27 (compressive)	23 (compressive)	
NbMoTaNi	SLM	1350.19 (compressive)	1356.19 (compressive)	11 (compressive)	
NbMoTaTi_0.5_Ni^0^.^5^	SLM	1750.46 (compressive)	2277.79 (compressive)	15 (compressive)	
NbMoTaTi_0.5_Ni_0.5_ (600 °C)	SLM	1279.34 (compressive)	1669.75 (compressive)	28.42 (compressive)	
NbMoTaTi_0.5_Ni_0.5_ (800 °C)	SLM	756.92 (compressive)	1033.63	28 (compressive)	
NbMoTaTi_0.5_Ni_0.5_ (1000 °C)	SLM	554.61 (compressive)	651.36 (compressive)	11 (compressive)	
AlCrFeNiV	SLM	651.36	1057.21	30.3	[142]
AlCoCrFeNi (0°)	SEBM	1015.0 ± 52.5 (compressive)	1668.3 ± 71.5 (compressive)	26.4 ± 6.7 (compressive)	[147]
AlCoCrFeNi (90°)	SEBM	944.0 ± 55.4 (compressive)	1447.0 ± 135.8 (compressive)	14.5 ± 5.3 (compressive)	
AlCoCrFeNi	SEBM	1015 ± 53 (compressive)	1668 ± 72 (compressive)	26.4 ± 6.7 (compressive)	[149]
AlCoCrFeNi	SEBM	769 ± 12.7	1073.5 ± 21.3	1.2 ± 0.2	[146]
AlCoCrFeNi	SEBM	–	312.6 ± 114.5	0	
CoCrFeNiMn	SEBM	205 ± 3	497 ± 2	63 ± 1	[148]
Co_1.5_CrFeNi_1.5_Ti_0.5_Mo_0.1_	SEBM		900	4	[151]
CoCrFeNiMn (Testing temperature: 25 °C)	DED(LAAM)	518 ± 3	660 ± 5	19.8 ± 1	[153]
CoCrFeNiMn (Testing temperature: 0 °C)	DED(LAAM)	565 ± 3	703 ± 3	28.9 ± 2	
CoCrFeNiMn (Testing temperature: −130 °C)	DED(LAAM)	710 ± 4	850 ± 2	40.2 ± 1	
CrMnFeCoNi	DED	320.7	531.7	31.9	[156]
CrMnFeCoNi	DED + 1 LSP	427.4	570.7	40.1	
CrMnFeCoNi	DED + 5 LSP	489.8	639.9	61	
FeCoCrNiMn	DED	330	630	55	[155]
Al_0.3_CoCrFeNi	DED	410		28	[163]
Al_0.3_CoCrFeNi	DED + 500 °C for 100 h	500		28	
Al_0.3_CoCrFeNi	DED + 620 °C for 50 h	630		18	
AlCoCrFeNi_2.1_ (X direction)	DED (LENS)	567 ± 41 (compressive)		≥16 (compressive)	[164]
AlCoCrFeNi_2.1_ (Z direction)	DED (LENS)	678 ± 19 (compressive)		≥16 (compressive)	
AlCoCrFeNi_2.1_ (Testing temperature: 400 °C)	DED (LENS)	711 ± 23 (compressive)		37 (compressive)	
AlCoCrFeNi_2.1_ (Testing temperature: 600 °C)	DED (LENS)	676 ± 8 (compressive)		45 (compressive)	
AlCoCrFeNi_2.1_ (Testing temperature: 700 °C)	DED (LENS)	548 ± 35 (compressive)		45 (compressive)	
AlCoCrFeNi_2.1_ (Testing temperature: 800 °C)	DED (LENS)	309 ± 17 (compressive)		45 (compressive)	
CrMnFeCoNi	DED (LENS)	517		26	[167]
CoCrFeMnNi	DED (LENS)	424	651.3	47.9	[168]
CoCrFeMnNi	DED (LENS) + 1100 °C for 1 h	232.2	647.1	58.3	
CoCrFeMnNi	DED (laser 3D printing)	448	620	57	[159]
CrMnFeCoNi	DED (LAM)	564	891	36	[194]
CrMnFeCoNi	DED (LAM)	352.5	540	26.1	
FeCrCoMnNi (laser power: 600 W)	DED (LAM)	346	566	26	[195]
FeCrCoMnNi (laser power: 800 W)	DED (LAM)	298	529	23	
FeCrCoMnNi (laser power: 1000 W)	DED (LAM)	307	547	30	
CrMnFeCoNi (testing temperature: 77 K)	DED (LMD)	402	878	95%	[175]
CrMnFeCoNi (testing temperature: 200 K)	DED (LMD)	304	610	73%	
CrMnFeCoNi (testing temperature: 293 K)	DED (LMD)	290	535	55%	
CrMnFeCoNi	DED (LMD)	300	550	50	[177]
CrMnFeCoNi + 5 wt% WC	DED (LMD)	502	776	37	
CrMnFeCoNi + 10 wt% WC	DED (LMD)	675	845	9	
FeCoCrNi	DED (LMD)	318.89	440.69	8.56	[179]
FeCoCrNi + FeCoCrNiAl-laminated HEA	DED (LMD)	383.42	533.39	10.61	
CoCrFeNiMo_0.2_ (laser power: 1000 W; testing temperature: 293 K)	DED (LMD)		532	37	[181]
CoCrFeNiMo_0.2_ (laser power: 1200 W; testing temperature: 293 K)	DED (LMD)		557	47	
CoCrFeNiMo_0.2_ (laser power: 1400 W; testing temperature: 293 K)	DED (LMD)		560	51	
CoCrFeNiMo_0.2_ (laser power: 1400 W; testing temperature: 77 K)	DED (LMD)		928	60	
Al_0.3_CoCrFeNi	DED (LMD)	476.9 ± 6.4	501.8 ± 2.9	0.60 ± 0.07	[182]
Al_0.3_CoCrFeNi	DED (LMD + annealed at 650 °C for 5 h)	373.5 ± 6.8	473.7 ± 6.4	2.96 ± 0.10	
CrMnFeCoNi	DED (LMD)	300	550	50	[184]
CrMnFeCoNi + 2.5 wt%TiC	DED (LMD)	330	610	47	
CrMnFeCoNi + 5 wt%TiC	DED (LMD)	385	723	32	
AlCoCrFeNi_2.1_	PAAM	356	719	27	[152]
AlCoCrFeNi_2.1_	PAAM	336	414	3	
AlCoCrFeNi_2.1_	PAAM	388	508	3	
CoCrFeNi	PPA-AM	141.8 ± 8.9 (compressive)			[196]
CoCrFeNi(SiC)_0.1_	PPA-AM	336.6 ± 10.61 (compressive)			
CoCrFeNi(SiC)_0.3_	PPA-AM	584.2 ± 12.3 (compressive)	2499.4 ± 20.6 (compressive)	48.5 ± 1.0 (compressive)	
CoCrFeNi(SiC)_0.5_	PPA-AM	712.7 ± 26.0 (compressive)	2155.2 ± 82.1 (compressive)	36.5 ± 2.8 (compressive)	
AlCoCrFeNi	BJT + annealed at 1000 °C	1203 ± 22 (compressive)	1996 ± 45 (compressive)	32.25 ± 2.5 (compressive)	[199]
AlCoCrFeNi	BJT + annealed at 1200 °C	1461 ± 23 (compressive)	2272 ± 48 (compressive)	31.46 ± 2.1 (compressive)	
FeCoNiCr	BJT + sintered at 1410 °C	134 ± 13	280 ± 19	16 ± 5	[201]
FeCoNiCr	BJT + sintered at 1410 °C and followed by HIP	133 ± 15	286 ± 21	16 ± 4	
FeCoNiCr	BJT + sintered at 1420 °C	132 ± 19	319 ± 15	25 ± 4	
FeCoNiCr	BJT + sintered at 1420 °C and followed by HIP	133 ± 16	322 ± 12	25 ± 7	
FeCoNiCr	BJT + sintered at 1430 °C	139 ± 17	335 ± 11	33 ± 5	
FeCoNiCr	BJT + sintered at 1430 °C and followed by HIP	143 ± 13	451 ± 9	49 ± 3	
FeCoNiCr	BJT + sintered at 1440 °C	181 ± 14	516 ± 17	52 ± 3	
FeCoNiCr	BJT + sintered at 1440 °C and followed by HIP	203 ± 17	547 ± 8	55 ± 2	

## Data Availability

No new data were created or analyzed in this study. Data sharing is not applicable to this article.

## Abbreviations

**Abbreviations**	**Full Name**
HEA	high-entropy alloy
AM	additive manufacturing
CAD	computer-aided design
SLM	selective laser melting
SLS	selective laser sintering
SEBM	selective electron beam melting
DED	directed energy deposition
BJT	binder jetting
DIW	direct ink writing
AFSD	additive friction stir deposition
BCC	body-centered cubic
FCC	face-centered cubic
HCP	hexagonal-closed packed
3D	three-dimensional
PBF	powder bed fusion
SHL	sheet lamination
VPP	vat photopolymerization
SL	stereolithography
SFF	solid freeform fabrication
3DP	3D printing technology
ME	material extrusion
SLA	stereolithography apparatus
DLP	digital light processing
LOM	laminated object manufacturing
PLA	polylactic acid
ABS	acrylonitrile butadiene styrene
PET	polyethylene terephthalate
PC	polycarbonate
LPBF	laser powder bed fusion
HAGB	high-angle grain boundary
SFE	stacking fault energy
GND	geometrically necessary dislocation
EPBF	electron powder bed fusion
PAAM	powder-bed arc additive manufacturing
LDC	laser direct casting
LMD	laser metal deposition
DMD	direct metal deposition
LC	laser consolidation
LMF	laser metal forming
LRF	laser rapid forming
LENS	laser engineered net shaping
LMD	laser metal deposition
DLF	directed laser fabrication
DLD	direct laser deposition
LF	laser forming
LAM	laser additive manufacturing
LBFEM	laser-based free entity manufacturing
LBFFF	laser-based free-form fabrication
LSF	laser solid forming
WLAM	wire laser additive manufacturing,
PAAM	powder-bed arc additive manufacturing
LAAM	laser aided additive manufacturing
WAAM	wire arc additive manufacturing
PPAAM	powder plasma arc additive manufacturing
KS	Kurdjumov–Sachs
PCT	pressure–composition–temperature
HIP	hot isostatic pressing
PREP	plasma rotating electrode process
LSP	laser shock peening
SCC	stress corrosion cracking
